# Discovery of a rich gene pool of bat SARS-related coronaviruses provides new insights into the origin of SARS coronavirus

**DOI:** 10.1371/journal.ppat.1006698

**Published:** 2017-11-30

**Authors:** Ben Hu, Lei-Ping Zeng, Xing-Lou Yang, Xing-Yi Ge, Wei Zhang, Bei Li, Jia-Zheng Xie, Xu-Rui Shen, Yun-Zhi Zhang, Ning Wang, Dong-Sheng Luo, Xiao-Shuang Zheng, Mei-Niang Wang, Peter Daszak, Lin-Fa Wang, Jie Cui, Zheng-Li Shi

**Affiliations:** 1 CAS Key Laboratory of Special Pathogens and Biosafety, Center for Emerging Infectious Diseases of Wuhan Institute of Virology, Chinese Academy of Sciences, Wuhan, China; 2 Yunnan Institute of Endemic Diseases Control and Prevention, Dali, China; 3 Dali University, Dali, China; 4 EcoHealth Alliance, New York, New York, United States of America; 5 Programme in Emerging Infectious Diseases, Duke-NUS Medical School, Singapore; Charite Universitatsmedizin Berlin, GERMANY

## Abstract

A large number of SARS-related coronaviruses (SARSr-CoV) have been detected in horseshoe bats since 2005 in different areas of China. However, these bat SARSr-CoVs show sequence differences from SARS coronavirus (SARS-CoV) in different genes (S, ORF8, ORF3, *etc*) and are considered unlikely to represent the direct progenitor of SARS-CoV. Herein, we report the findings of our 5-year surveillance of SARSr-CoVs in a cave inhabited by multiple species of horseshoe bats in Yunnan Province, China. The full-length genomes of 11 newly discovered SARSr-CoV strains, together with our previous findings, reveals that the SARSr-CoVs circulating in this single location are highly diverse in the S gene, ORF3 and ORF8. Importantly, strains with high genetic similarity to SARS-CoV in the hypervariable N-terminal domain (NTD) and receptor-binding domain (RBD) of the S1 gene, the ORF3 and ORF8 region, respectively, were all discovered in this cave. In addition, we report the first discovery of bat SARSr-CoVs highly similar to human SARS-CoV in ORF3b and in the split ORF8a and 8b. Moreover, SARSr-CoV strains from this cave were more closely related to SARS-CoV in the non-structural protein genes ORF1a and 1b compared with those detected elsewhere. Recombination analysis shows evidence of frequent recombination events within the S gene and around the ORF8 between these SARSr-CoVs. We hypothesize that the direct progenitor of SARS-CoV may have originated after sequential recombination events between the precursors of these SARSr-CoVs. Cell entry studies demonstrated that three newly identified SARSr-CoVs with different S protein sequences are all able to use human ACE2 as the receptor, further exhibiting the close relationship between strains in this cave and SARS-CoV. This work provides new insights into the origin and evolution of SARS-CoV and highlights the necessity of preparedness for future emergence of SARS-like diseases.

## Author summary

Increasing evidence has been gathered to support the bat origin of SARS coronavirus (SARS-CoV) in the past decade. However, none of the currently known bat SARSr-CoVs is thought to be the direct ancestor of SARS-CoV. Herein, we report the identification of a diverse group of bat SARSr-CoVs in a single cave in Yunnan, China. Importantly, all of the building blocks of SARS-CoV genome, including the highly variable S gene, ORF8 and ORF3, could be found in the genomes of different SARSr-CoV strains from this single location. Based on the analysis of full-length genome sequences of the newly identified bat SARSr-CoVs, we speculate that the direct ancestor of SARS-CoV may have arisen from sequential recombination events between the precursors of these bat SARSr-CoVs prior to spillover to an intermediate host. In addition, we found bat SARSr-CoV strains with different S proteins that can all use the receptor of SARS-CoV in humans (ACE2) for cell entry, suggesting diverse SARSr-CoVs capable of direct transmission to humans are circulating in bats in this cave. Our current study therefore offers a clearer picture on the evolutionary origin of SARS-CoV and highlights the risk of future emergence of SARS-like diseases.

## Introduction

Severe Acute Respiratory Syndrome (SARS) is a severe emerging viral disease with high fatality characterized by fever, headache and severe respiratory symptoms including cough, dyspnea and pneumonia [1]. Due to its high transmissibility among humans, after its first emergence in southern China in late 2002, it rapidly led to a global pandemic in 2003 and was marked as one of the most significant public health threats in the 21^st^ century [2,3]. The causative agent, SARS coronavirus (SARS-CoV), has been previously assigned to group 2b CoV and is now a member of the lineage B of genus *Betacoronavirus* in the family *Coronaviridae* [4]. It shares similar genome organization with other coronaviruses, but exhibits a unique genomic structure which includes a number of specific accessory genes, including ORF3a, 3b, ORF6, ORF7a, 7b, ORF8a, 8b and 9b [5,6].

Masked palm civets (*Paguma larvata*) were initially hypothesized to be the animal origin of SARS-CoV [7,8]. However, since a large number of genetically diverse SARS-related coronaviruses (SARSr-CoV) have been detected in multiple species of horseshoe bats (genus *Rhinolophus*) from different areas of China and Europe in the aftermath of SARS, it is prevailingly considered that SARS-CoV originated in horseshoe bats with civets acting as the intermediate amplifying and transmitting host [9–16]. Recently we have reported four novel SARSr-CoVs from Chinese horseshoe bats that shared much higher genomic sequence similarity to the epidemic strains, particularly in their S gene, of which two strains (termed WIV1 and WIV16) have been successfully cultured *in vitro* [17,18]. These newly identified SARSr-CoVs have been demonstrated to use the same cellular receptor (angiotensin converting enzyme-2 [ACE-2]) as SARS-CoV does and replicate efficiently in primary human airway cells [17–19].

Despite the cumulative evidence for the emergence of SARS-CoV from bats, all bat SARSr-CoVs described so far are clearly distinct from SARS-CoV in the S gene and/or one or more accessory genes such as ORF3 and ORF8, suggesting they are likely not the direct ancestor of SARS-CoV. Thus a critical gap remains in our understanding of how and where SARS-CoV originated from bat reservoirs. Previously, we reported a number of bat SARSr-CoVs with diverse S protein sequences from a single cave in Yunnan Province, including the four strains mentioned above most closely related to SARS-CoV [17,18]. Here we report the latest results of our 5-year longitudinal surveillance of bat SARSr-CoVs in this single location and systematic evolutionary analysis using full-length genome sequences of 15 SARSr-CoV strains (11 novel ones and 4 from previous studies). Efficiency of human ACE2 usage and the functions of accessory genes ORF8 and 8a were also evaluated for some of the newly identified strains.

## Results

### Continued circulation of diverse SARSr-CoVs in bats from a single location

We have carried out a five-year longitudinal surveillance (April 2011 to October 2015) on SARSr-CoVs in bats from a single habitat in proximity to Kunming city, Yunnan province, China, which was mainly inhabited by horseshoe bats. A total of 602 alimentary specimens (anal swabs or feces) were collected and tested for the presence of CoVs by a Pan-CoV RT-PCR targeting the 440-nt RdRp fragment that is conserved among all known α- and β-CoVs [20]. In total, 84 samples tested positive for CoVs. Sequencing of the PCR amplicons revealed the presence of SARSr-CoVs in the majority (64/84) of the CoV-positive samples (Table 1). Host species identification by amplification of either *Cytb* or *ND1* gene suggested that most (57/64) of the SARSr-CoV positive samples were from *Rhinolophus sinicus*, while the remaining 7 samples were from *Rhinolophus ferrumequinum*, *Rhinolophus affinis* and from *Aselliscus stoliczkanus* which belongs to the family *Hipposideridae*.

**Table 1:** Summary of SARSr-CoV detection in bats from a single habitat in Kunming, Yunnan.

Sampling time	Sample type	Sample Numbers				SARSr-CoV + bat species (No.)
		Total	CoV +	SARSr-CoV +		
April, 2011	anal swab	14	1		1	*R*. *sinicus* (1)
October, 2011	anal swab	8	3		3	*R*. *sinicus* (3)
May, 2012	anal swab & feces	54	9		4	*R*. *sinicus* (4)
September, 2012	feces	39	20		19	*R*. *sinicus* (16)
						*R*. *ferrumequinum* (3)
April, 2013	feces	52	21		16	*R*. *sinicus* (16)
July, 2013	anal swab & feces	115	9		8	*R*. *sinicus* (8)
May, 2014	feces	131	8		4	*A*. *stoliczkamus* (3)
						*R*. *affinis* (1)
October, 2014	anal swab	19	4		4	*R*. *sinicus* (4)
May, 2015	feces	145	3		0	
October, 2015	anal swab	25	6		5	*R*. *sinicus* (5)
***Total***		***602***	***84***		***64***	***R (61) A (3)***

Based on the preliminary analysis of the partial RdRp sequences, all of the 64 bat SARSr-CoV sequences showed high similarity among themselves and with other reported bat SARSr-CoVs and SARS-CoVs from humans and civets. To understand the genetic diversity of these bat SARSr-CoVs, the most variable region of the SARSr-CoV S gene, corresponding to the receptor-binding domain (RBD) of SARS-CoV, were amplified and sequenced. Due to low viral load in some samples, RBD sequences were successfully amplified only from 49 samples. These RBD sequences displayed high genetic diversity and could be divided into two large clades, both of which included multiple genotypes. Clade 1 strains shared an identical size and higher amino acid (aa) sequence identity with SARS-CoV RBD, while clade 2 had a shorter size than SARS-CoV S due to two deletions (5 and 12–13 aa, respectively) (S1 Fig). Co-infections by two strains of different clades were detected in two samples, Rs3262 and Rs4087 (S1 Fig).

### Genomic characterization of the novel SARSr-CoVs

**Fig 1:**
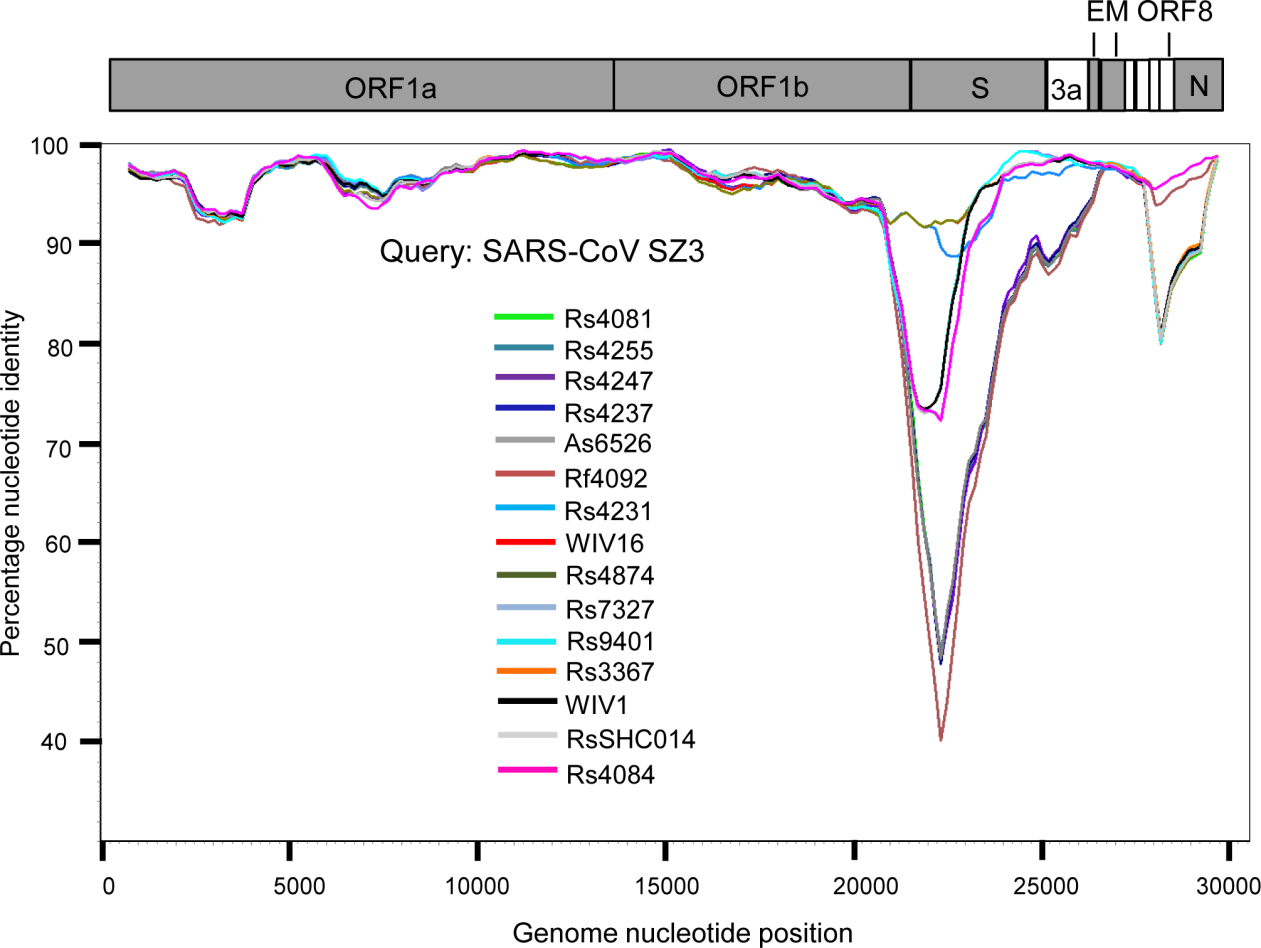
Similarity plot based on the full-length genome sequence of civet SARS CoV SZ3. Full-length genome sequences of all SARSr-CoV detected in bats from the cave investigated in this study were used as reference sequences. The analysis was performed with the Kimura model, a window size of 1500 base pairs and a step size of 150 base pairs.

Based on the diversity of RBD sequences, 11 novel SARSr-CoV strains named by abbreviation of bat species and sample ID (Rs4081, Rs4084, Rs4231, Rs4237, Rs4247, Rs4255, Rs4874, Rs7327, Rs9401, Rf4092 and As6526) were selected for full-length genomic sequencing based on sample abundance, genotype of RBD as well as sampling time. For each RBD genotype and each time of sampling, at least one representative strain was selected. The genome size of these novel SARSr-CoVs ranged from 29694 to 30291 nucleotides (nt). This gave a total of 15 full-length genomes of bat SARSr-CoVs from this single location (13 from *R*.*sinicus*, and one each from *R*. *ferrumequinum* and *A*. *stoliczkanus*), including our previously reported strains, Rs3367, RsSHC014, WIV1 and WIV16 [17,18]. The genomes of all 15 SARSr-CoVs circulating in this single cave shared 92.0% to 99.9% nt sequence identity. The overall nt sequence identity between these SARSr-CoVs and human and civet SARS-CoVs is 93.2% to 96%, significantly higher than that observed for bat SARSr-CoVs reported from other locations in China (88–93%) [9,10,12,14,21,22]. The genome sequence similarity among the 15 SARSr-CoVs and SARS-CoV SZ3 strain was examined by Simplot analysis (Fig 1). The 15 SARSr-CoVs are highly conserved and share a uniformly high sequence similarity to SARS-CoV in the non-structural gene ORF1a (96.6% to 97.1% nt sequence identity, 98.0% to 98.3% aa sequence identity) and ORF1b (96.1% to 96.6% nt sequence identity, 99.0% to 99.4% aa sequence identity). In contrast, a considerable genetic diversity is shown in the S gene (corresponding to SZ3 genome position 21477 to 25244) and ORF8 (corresponding to SZ3 genome position 27764 to 28132) (Fig 1).

**Fig 2:**
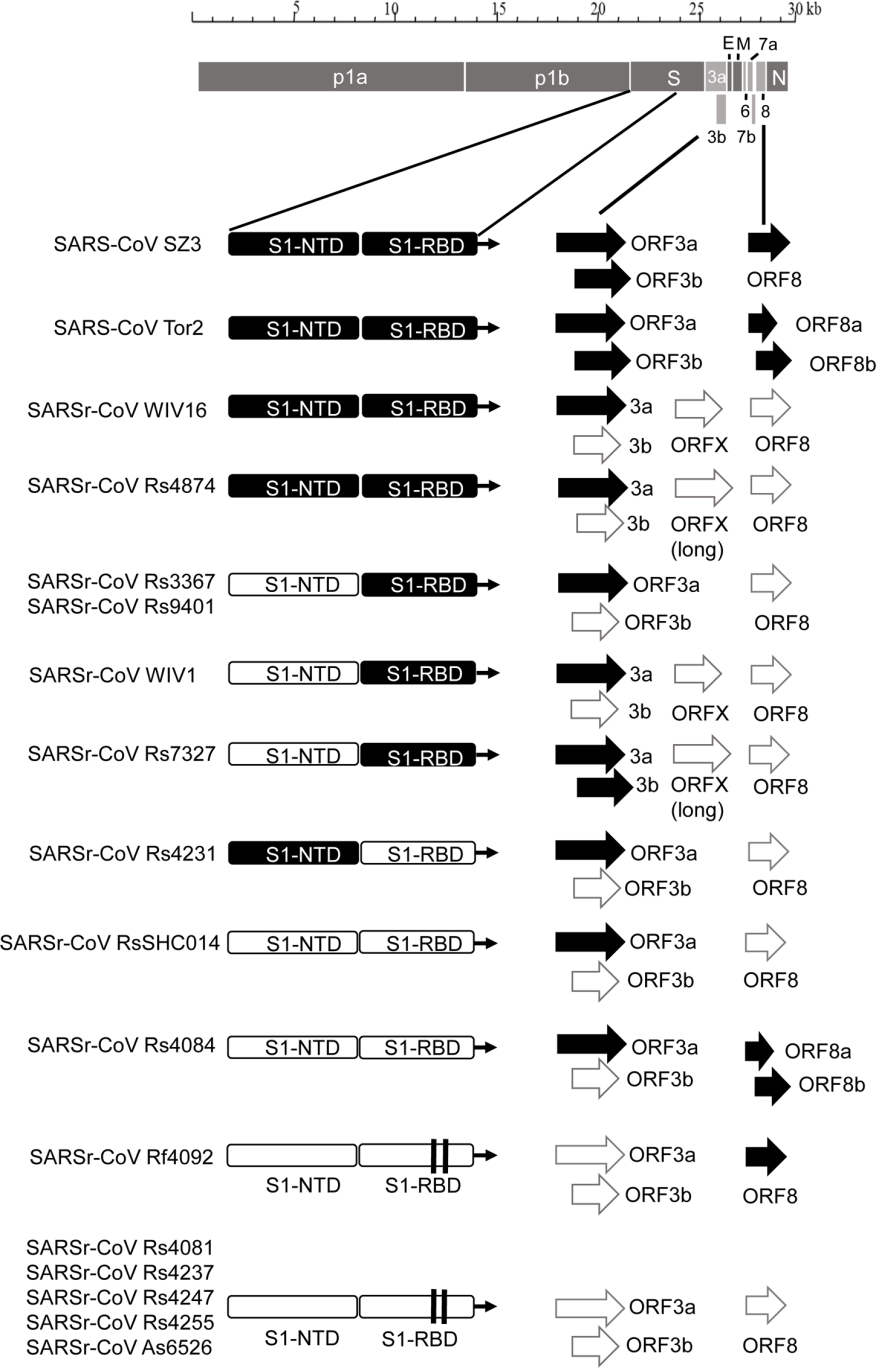
Schematic diagram illustrating the genomic regions or ORFs with most variation between different SARS-CoV and SARSr-CoV isolates. Coding regions of the N-terminal domain (NTD) and receptor-binding domain (RBD) of the spike protein, ORF3a/b and ORF8 (8a/b) in bat SARSr-CoV genomes highly similar to those in SARS CoV genome are indicated with black boxes or arrows while the hollow boxes or arrows represent corresponding regions with less sequence similarity to those of SARS-CoV. The deletions in the RBD of some SARSr-CoVs are indicated by two vertical lines.

The 11 novel SARSr-CoVs identified from this single location generally shared similar genome organization with SARS-CoV and other bat SARSr-CoVs. In our previous study, we identified an additional ORF termed ORFx present between ORF6 and ORF7 in strain WIV1 and WIV16 [18,23]. In this study, ORFx was also found in the genomes of Rs7327 and Rs4874. Compared with that of WIV1 and WIV16, the length of ORFx in Rs7327 and Rs4874 was extended to 510 nt due to a deletion of 2 nt in a poly-T sequence that resulted in a shift of reading frame (Fig 2 and S2 Fig).

### Co-circulation of different bat SARSr-CoVs with S, ORF8 and ORF3 sequences similar to those in SARS-CoV at a single location

**Fig 3:**
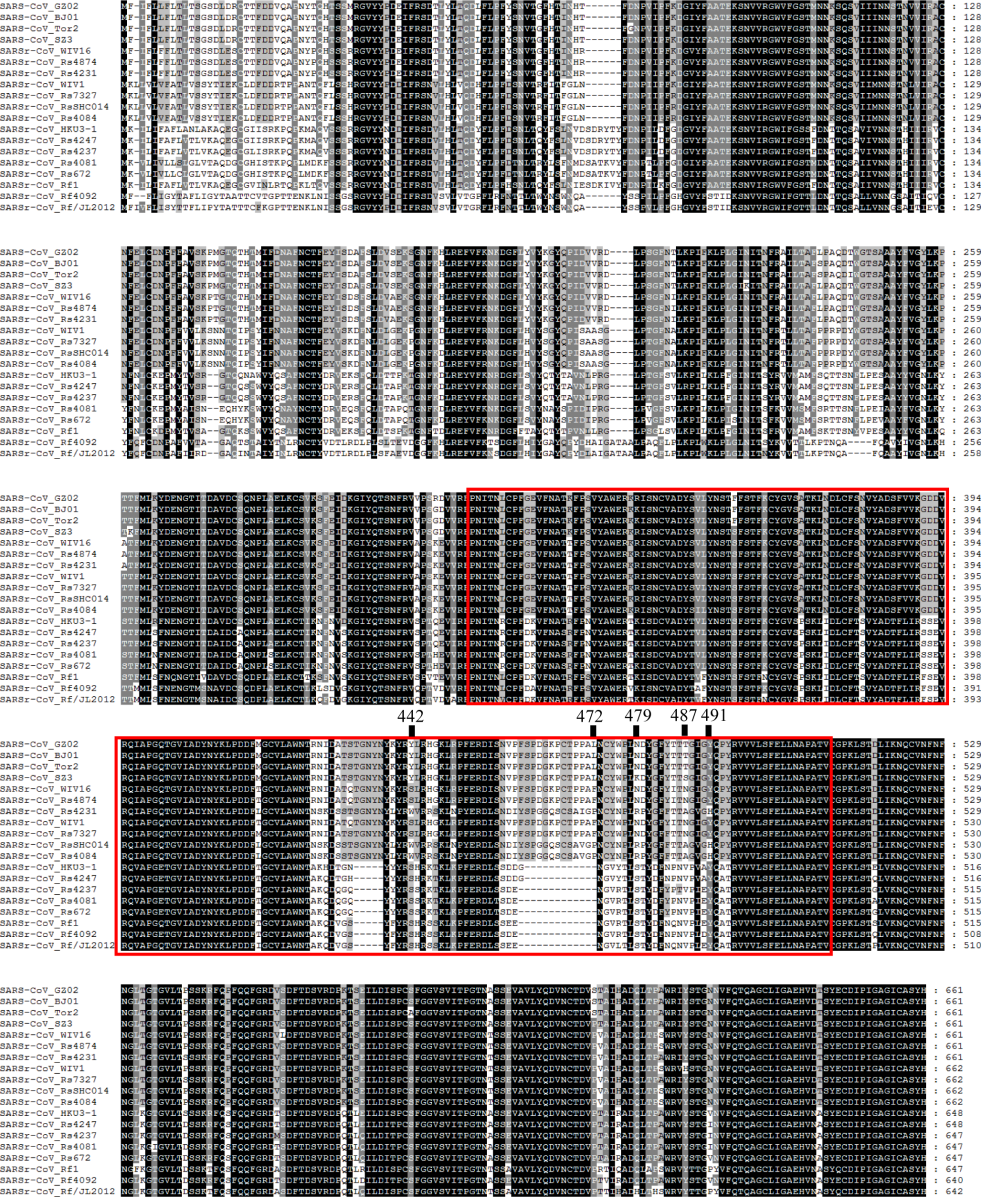
Amino acid sequence comparison of the S1 subunit (corresponding to aa 1–660 of the spike protein of SARS-CoV). The receptor-binding domain (aa 318–510) of SARS-CoV and the homologous region of bat SARSr-CoVs are indicated by the red box. The key aa residues involved in the interaction with human ACE2 are numbered on top of the aligned sequences. SARS-CoV GZ02, BJ01 and Tor2 were isolated from patients in the early, middle and late phase, respectively, of the SARS outbreak in 2003. SARS-CoV SZ3 was identified from civets in 2003. SARSr-CoV Rs 672 and YN2013 were identified from *R*. *sinicus* collected in Guizhou and Yunnan Province, respectively. SARSr-CoV Rf1 and JL2012 were identified from *R*. *ferrumequinum* collected in Hubei and Jilin Province, respectively. WIV1, WIV16, RsSHC014, Rs4081, Rs4084, Rs4231, Rs4237, Rs4247, Rs7327 and Rs4874 were identified from *R*.*sinicus*, and Rf4092 from *R*. *ferrumequinum* in the cave surveyed in this study.

The primary difference between SARS-CoV and most bat SARSr-CoVs is located in S gene. The S protein is functionally divided into two subunits, denoted S1 and S2, which is responsible for receptor binding and cellular membrane fusion, respectively. S1 consists of two domains, the N-terminal domain (NTD) and C-terminal domain (CTD) which is also known as the RBD in SARS-CoV [24]. SARS-CoV and bat SARSr-CoVs share high sequence identity in the S2 region in contrast to the S1 region. Among the 15 SARSr-CoVs identified from bats in the surveyed cave, six strains with deletions in their RBD regions (Rs4081, Rs4237, Rs4247, Rs4255, Rf4092 and As6526) showed 78.2% to 80.2% aa sequence identity to SARS-CoV in the S protein, while the other nine strains without deletions were much more closely related to SARS-CoV, with 90.0% (Rs4084) to 97.2% (Rs4874) aa sequence identity. These nine SARSr-CoVs can be further divided into four genotypes according to their S1 sequences (Fig 2): RsSHC014/Rs4084 showed more genetic differences from SARS-CoV in both NTD and RBD regions; The RBD sequences of SARSr-CoV Rs7327, Rs9401 and previously reported WIV1/Rs3367 closely resembled that of SARS-CoV. However, they were distinct from SARS-CoV but similar to RsSHC014 in NTD. In contrast, we found a novel SARSr-CoV, termed Rs4231, which shared highly similar NTD, but not RBD sequence with SARS-CoV (Figs 2 and 3). Its S protein showed 94.6% to 95% aa sequence identity to those of human and civet SARS-CoVs (S1 Table). Strains with both NTD and RBD highly homologous to those of SARS-CoV were also present in this cave. In addition to WIV16 which we described previously [18], Rs4874 was also found to have the S protein closest to SARS-CoV S (> 97% aa sequence identity) of all the bat SARSr-CoVs reported to date (Figs 2 and 3). In addition to the SARSr-CoVs subjected to full-length genome sequencing, we also obtained the RBD and NTD sequences from other samples collected in this cave. The sequences with high identity to SARS-CoV RBD were amplified from 10 more *R*. *sinicus* samples. SARSr-CoVs with this genotype of RBD were detected in different seasons throughout the five years. Strains containing the NTD similar to SARS-CoV were only found in 2013 (S2 Table).

**Fig 4:**
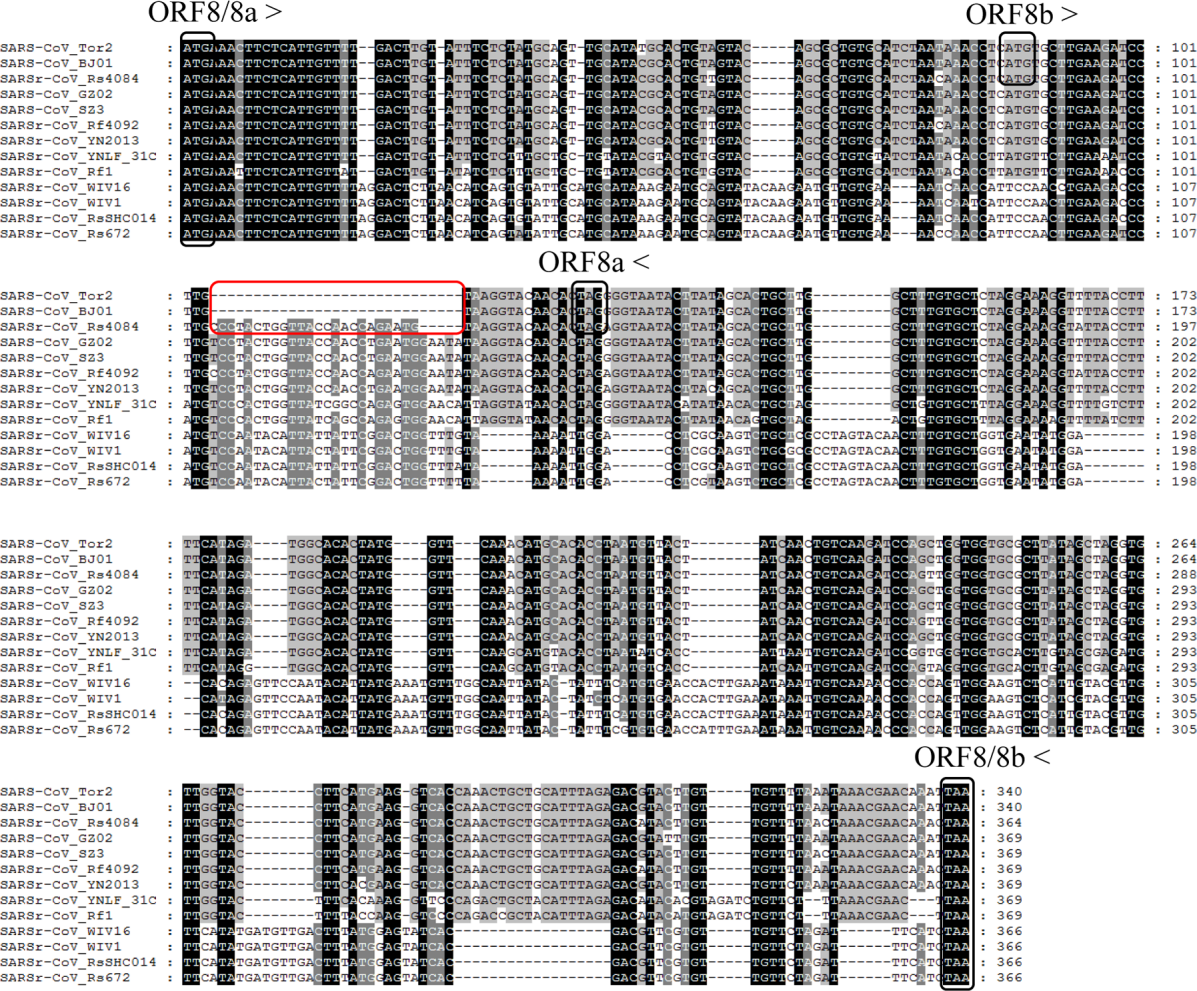
Alignment of nucleotide sequences of ORF8 or ORF8a/8b. The start codons and stop codons of ORF8, 8a and 8b are marked with black boxes and the forward and reverse arrows, respectively. The deletion responsible for the split ORF8a and 8b in human SARS-CoV BJ01, Tor2 and bat SARSr-CoV Rs4084 is marked with red boxes. See the legend for Fig 3 for the origin of various sequences used in this alignment.

ORF8 is another highly variable gene among different SARS-CoV and SARSr-CoV strains [25,26]. We aligned the ORF8 nt sequences of the representative SARSr-CoVs discovered in this surveillance with those of other SARSr-CoVs and SARS-CoVs (Fig 4). Though WIV16, WIV1, Rs4231 and RsSHC014 were genetically closer to SARS-CoV in S gene, they contained a single 366-nt ORF8 without the 29-nt deletion present in most human SARS-CoVs and showed only 47.1% to 51.0% nt sequence identity to human and civet SARS-CoVs. However, the ORF8 of strain Rf4092 from *R*. *ferrumequinum* exhibited high similarity to that of civet SARS-CoV. It possessed a single long ORF8 of the same length (369 nt) as that of civet SARS-CoV strain SZ3, with only 10 nt mutations and 3 aa mutations detected (Fig 4). Similar ORF8 sequences were also amplified from other 7 samples collected in the cave during 2011 to 2013, from both *R*. *ferrumequinum* and *R*. *sinicus* (S2 Table). The ORF8 of Rs4084 was highly similar to Rf4092’s but was split into two overlapping ORFs, ORF8a and ORF8b, due to a short 5-nt deletion (Figs 2 and 4). The position of start codons and stop codons of the two ORFs were consistent with those in most human SARS-CoV strains. Excluding the 8-aa insertion, Rs4084 and SARS-CoV strain BJ01 displayed identical aa sequence of ORF8a, and only three different aa residues were observed between their ORF8b (Fig 4). To our knowledge, Rs4084 was the first bat SARSr-CoV reported that resembled the late human SARS-CoVs in both ORF8 gene organization and sequence.

Another key difference between SARS-CoV and bat SARSr-CoV genomes is the ORF3 coding region [10,17,21]. We analyzed the ORF3a sequences amplified from 42 samples and found that most of the SARSr-CoVs closely related to SARS-CoV in the S gene shared higher ORF3a sequence similarity (96.4% to 98.9% aa identity) with SARS-CoV (S3 Fig and S2 Table). The ORF3b of SARS CoV, sharing a large part of its coding sequence with the ORF3a, encodes a 154-aa protein [27], but it is truncated to different extents at the C-terminal in previously described bat SARSr-CoVs including WIV1 and WIV16 (S4 Fig). In the current study, we identified a non-truncated ORF3b for the first time (Rs7327), which maintained the nuclear localization signal at its C-terminal. Moreover, it shared 98.1% aa sequence identity with SARS-CoV strain Tor2 with only three aa substitutions (S4 Fig). Thus, Rs7327 is the bat SARSr-CoV most similar to SARS-CoV in the ORF3 region known to date.

### Recombination analysis

**Fig 5:**
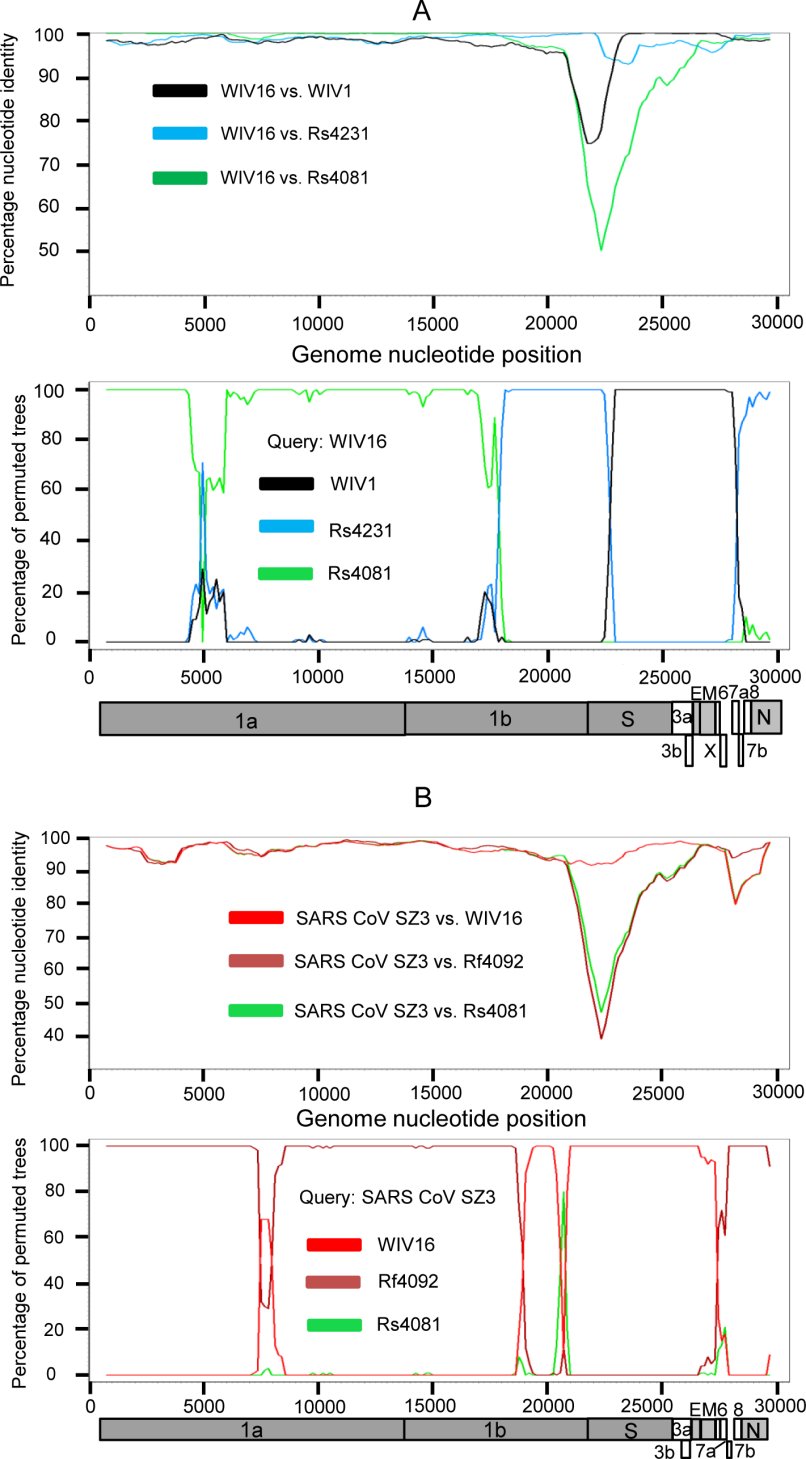
Detection of potential recombination events by similarity plot and boot scan analysis. (A) Full-length genome sequence of SARSr-CoV WIV16 was used as query sequence and WIV1, Rs4231 and Rs4081 as reference sequences. (B) Full-length genome sequence of SARS-CoV SZ3 was used as query sequence and SARSr-CoV WIV16, Rf4092 and Rs4081 as reference sequences. All analyses were performed with a Kimura model, a window size of 1500 base pairs, and a step size of 150 base pairs. The gene map of query genome sequences are used to position breakpoints.

The full-length genome sequences of all 15 SARSr-CoVs from the surveyed cave were screened for evidence of potential recombination events. Both similarity plot and bootscan analyses revealed frequent recombination events among these SARSr-CoV strains. It was suggested that WIV16, the closest progenitor of human SARS-CoV known to date [18], was likely to be a recombinant strain from three SARSr-CoVs harbored by bats in the same cave, namely WIV1, Rs4231 and Rs4081, with strong *P* value (<10^−30^). Breakpoints were identified at genome positions nt 18391, 22615 and 28160 (Fig 5A). In the genomic region between nt 22615 and 28160, which contained the region encoding the RBD and the S2 subunit of the S protein, WIV16 was highly similar to WIV1, sharing 99% sequence identity. In contrast, in the region between nt 18391 and 22615, which covered a part of ORF1b and the region encoding the NTD of the S gene, WIV16 showed substantially closer relationship to Rs4231. Meanwhile, the ORF1ab sequences upstream from nt 18391 of WIV16 displayed the highest genetic similarity (99.8% nt sequence identity) to that of Rs4081.

Evidence of recombination event was also detected in the genome of the novel SARSr-CoV Rs4084, which had a unique genome organization with split ORF8a and 8b. The previously reported strain RsSHC014 and the newly identified strain Rf4092 were suggested to be the major and minor parent of Rs4084, respectively (*P* value < 10^−80^). The breakpoint was located at nt 26796 (S5 Fig). In the region downstream of the breakpoint including ORF8, Rs4084 showed closet genetic relationship with Rf4092, sharing 98.9% nt sequence identity, while it shared the highest nt sequence identity (99.4%) with RsSHC014 in the majority of its genome upstream from the breakpoint.

When civet SARS-CoV SZ3 was used as the query sequence in similarity plot and bootscan analysis, evidence for recombination events was also detected (Fig 5B). In the region between the two breakpoints at the genome positions nt 21161 and nt 27766, including the S gene, closer genetic relationship between SZ3 and WIV16 was observed. However, from position nt 27766 towards the 3’ end of its genome, a notably close genetic relationship was observed between SZ3 and Rf4092 instead. Throughout the non-structural gene, moreover, SZ3 shared a similarly high sequence identity with WIV16 and Rf4092. It indicates that civet SARS-CoV was likely to be the descendent from a recombinant of the precursors of WIV16 and Rf4092, or that the SARSr-CoVs found in this cave, like WIV16 or Rf4092, may have been the descendants of the SARS-CoV lineage.

### Phylogenetic analysis

**Fig 6:**
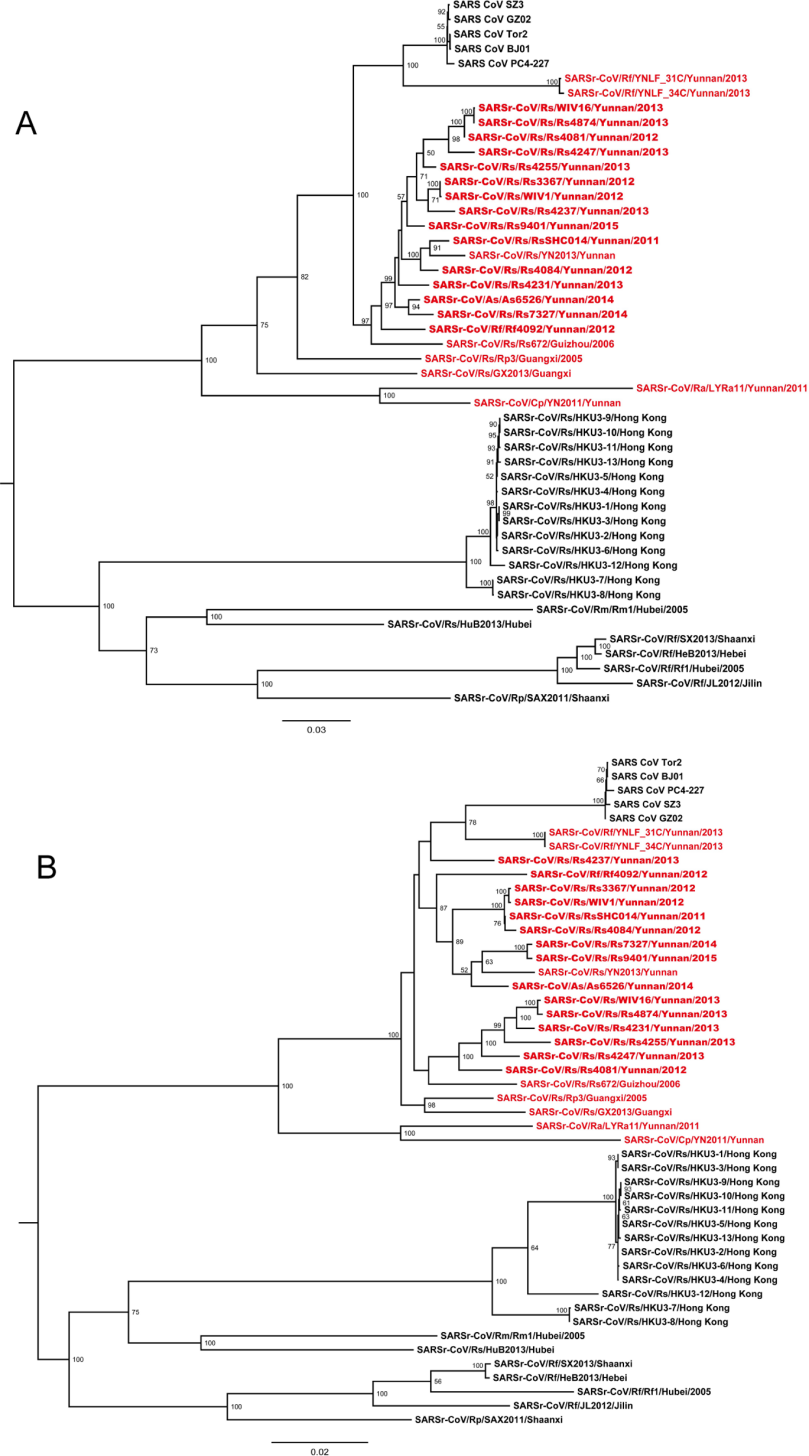
**Phylogenetic trees based on nucleotide sequences of ORF1a (A) and ORF1b (B).** The trees were constructed by the maximum likelihood method using the LG model with bootstrap values determined by 1000 replicates. Only bootstraps > 50% are shown. The scale bars represent 0.03 (A) and 0.02 (B) substitutions per nucleotide position. Rs, *Rhinolophus sinicus*; Rf, *Rhinolophus ferremequinum*; Rm, *Rhinolophus macrotis*; Ra, *Rhinolophus affinis*; Rp, *Rhinolophus pusillus*; As, *Aselliscus stoliczkanus*; Cp, *Chaerephon plicata*. SARSr-CoVs detected in bats from the single cave surveyed in this study are in bold. Sequences detected in southwestern China are indicated in red.

Phylogenetic trees were constructed using the nt sequences of nonstructural protein gene ORF1a and ORF1b. Unlike the high genetic diversity in the S gene, nearly all SARSr-CoVs from the bat cave we surveyed were closely clustered, and showed closer phylogenetic relationship to SARS-CoV than the majority of currently known bat SARSr-CoVs discovered from other locations, except YNLF_31C and 34C which were recently reported in greater horseshoe bats from another location in Yunnan [22] (Fig 6). The phylogeny of SARSr-CoVs in ORF1a and ORF1b appeared to be associated with their geographical distribution rather than with host species. Regardless of different host bat species, SARS-CoV and SARSr-CoVs detected in bats from southwestern China (Yunnan, Guizhou and Guangxi province) formed one clade, in which SARSr-CoV strains showing closer relationship to SARS-CoV were all from Yunnan. SARSr-CoVs detected in southeastern, central and northern provinces, such as Hong Kong, Hubei and Shaanxi, formed the other clade which was phylogenetically distant to human and civet SARS-CoVs (Fig 6 and S6 Fig).

### Rescue of bat SARSr-CoVs and virus infectivity experiments

**Fig 7:**
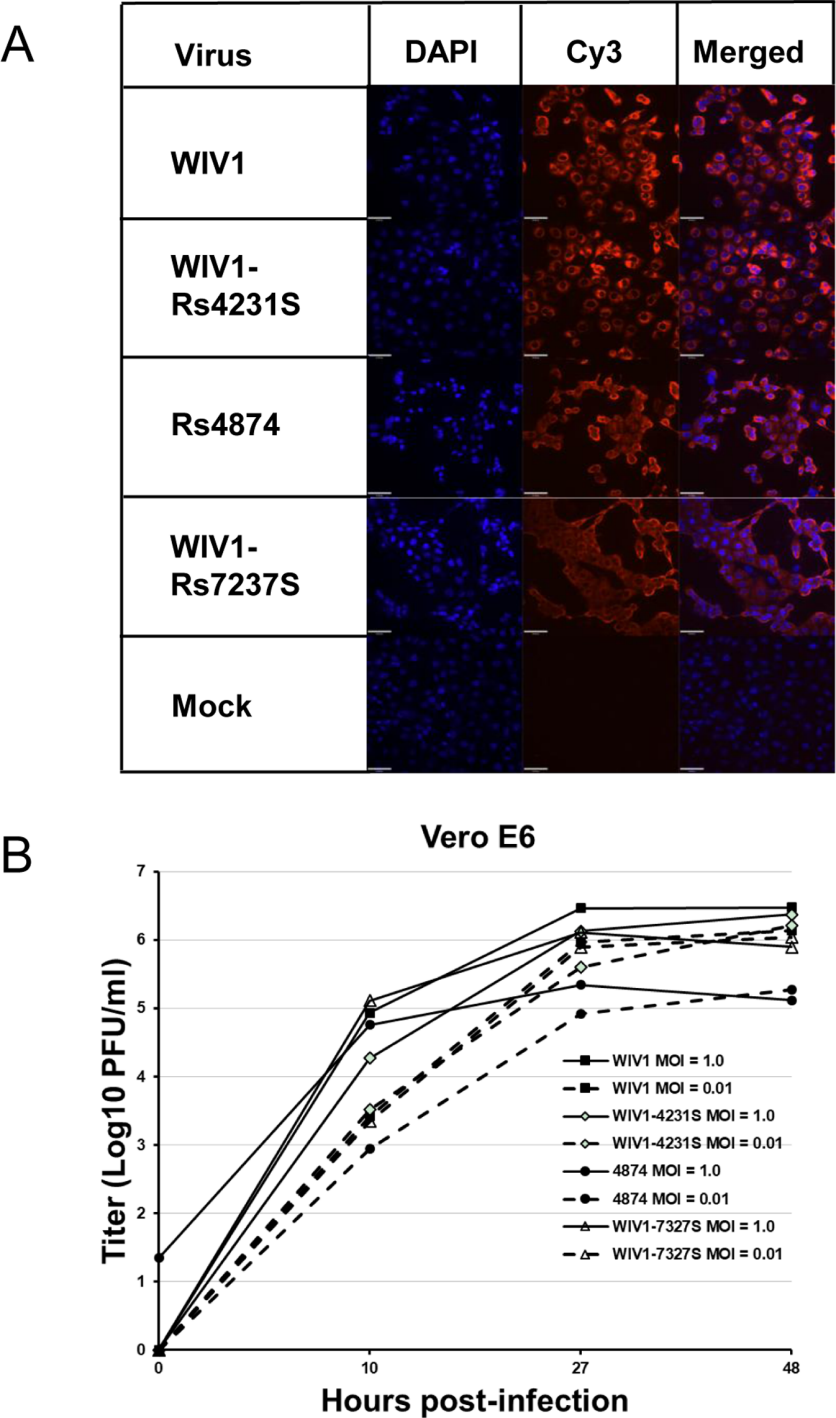
Infection of Vero E6 cells by bat SARSr-CoV WIV1, Rs4874, WIV1-Rs4231S and WIV1-Rs7327S. (A) The successful infection was confirmed by immunofluorescent antibody staining using rabbit antibody against the SARSr-CoV Rp3 nucleocapsid protein. The columns (from left to right) show staining of nuclei (blue), virus replication (red), and both nuclei and virus replication (merged double-stain images). (B) The growth curves in Vero E6 cells with a MOI of 1.0 and 0.01.

**Fig 8:**
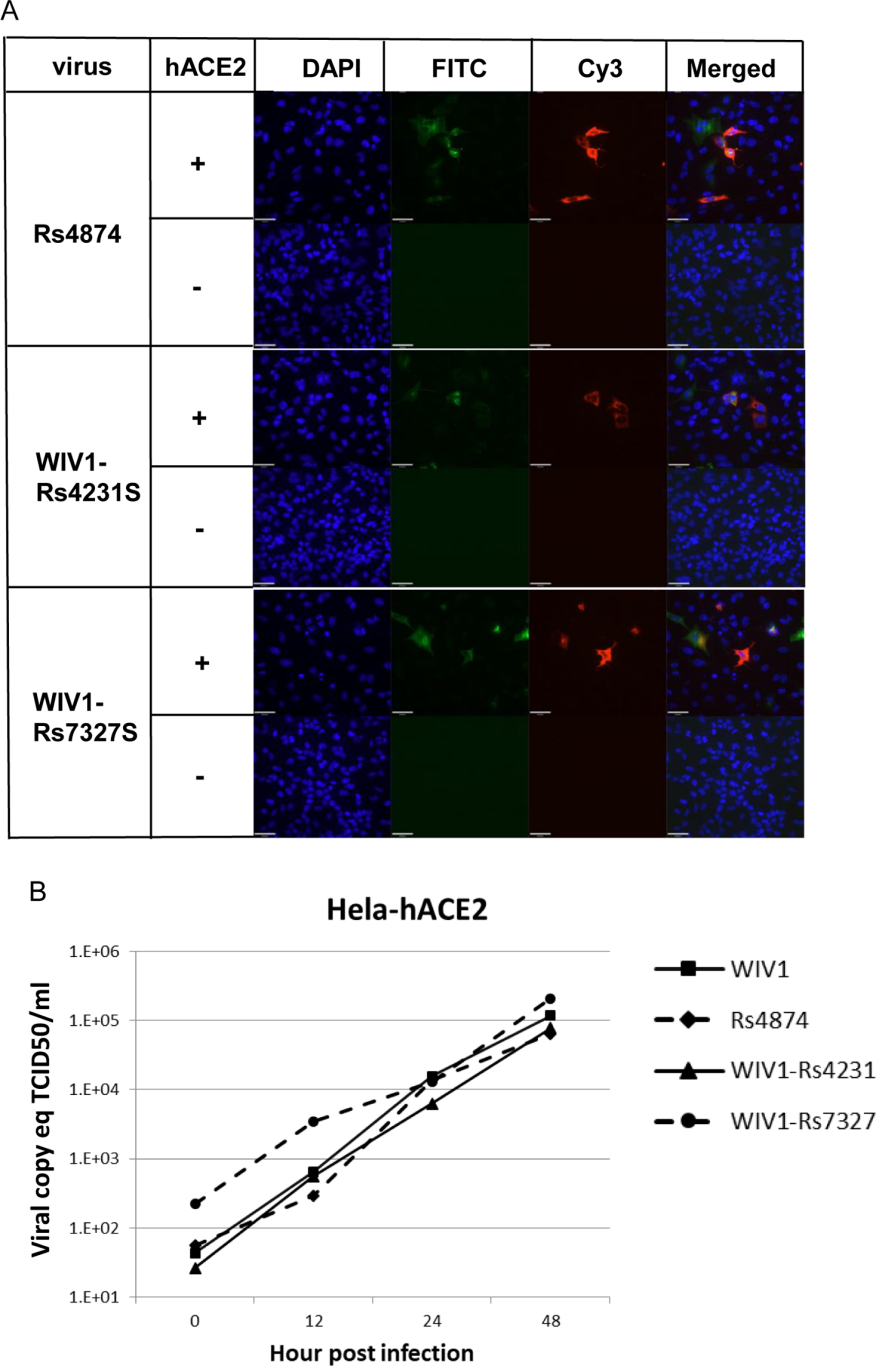
**Analysis of receptor usage by immunofluorescence assay (A) and real-time PCR (B).** Virus infectivity of Rs4874, WIV1-Rs4231S and WIV1-Rs7327S was determined in HeLa cells with and without the expression of human ACE2. ACE2 expression was detected with goat anti-human ACE2 antibody followed by fluorescein isothiocyanate (FITC)-conjugated donkey anti-goat IgG. Virus replication was detected with rabbit antibody against the SARSr-CoV Rp3 nucleocapsid protein followed by cyanine 3 (Cy3)-conjugated mouse anti-rabbit IgG. Nuclei were stained with DAPI (49,6-diamidino-2-phenylindole).The columns (from left to right) show staining of nuclei (blue), ACE2 expression (green), virus replication (red) and the merged triple-stained images, respectively.

In the current study, we successfully cultured an additional novel SARSr-CoV Rs4874 from a single fecal sample using an optimized protocol and Vero E6 cells [17]. Its S protein shared 99.9% aa sequence identity with that of previously isolated WIV16 and it was identical to WIV16 in RBD. Using the reverse genetics technique we previously developed for WIV1 [23], we constructed a group of infectious bacterial artificial chromosome (BAC) clones with the backbone of WIV1 and variants of S genes from 8 different bat SARSr-CoVs. Only the infectious clones for Rs4231 and Rs7327 led to cytopathic effects in Vero E6 cells after transfection (S7 Fig). The other six strains with deletions in the RBD region, Rf4075, Rs4081, Rs4085, Rs4235, As6526 and Rp3 (S1 Fig) failed to be rescued, as no cytopathic effects was observed and viral replication cannot be detected by immunofluorescence assay in Vero E6 cells (S7 Fig). In contrast, when Vero E6 cells were respectively infected with the two successfully rescued chimeric SARSr-CoVs, WIV1-Rs4231S and WIV1-Rs7327S, and the newly isolated Rs4874, efficient virus replication was detected in all infections (Fig 7). To assess whether the three novel SARSr-CoVs can use human ACE2 as a cellular entry receptor, we conducted virus infectivity studies using HeLa cells with or without the expression of human ACE2. All viruses replicated efficiently in the human ACE2-expressing cells. The results were further confirmed by quantification of viral RNA using real-time RT-PCR (Fig 8).

### Activation of activating transcription factor 6 (ATF6) by the ORF8 proteins of different bat SARSr-CoVs

**Fig 9:**
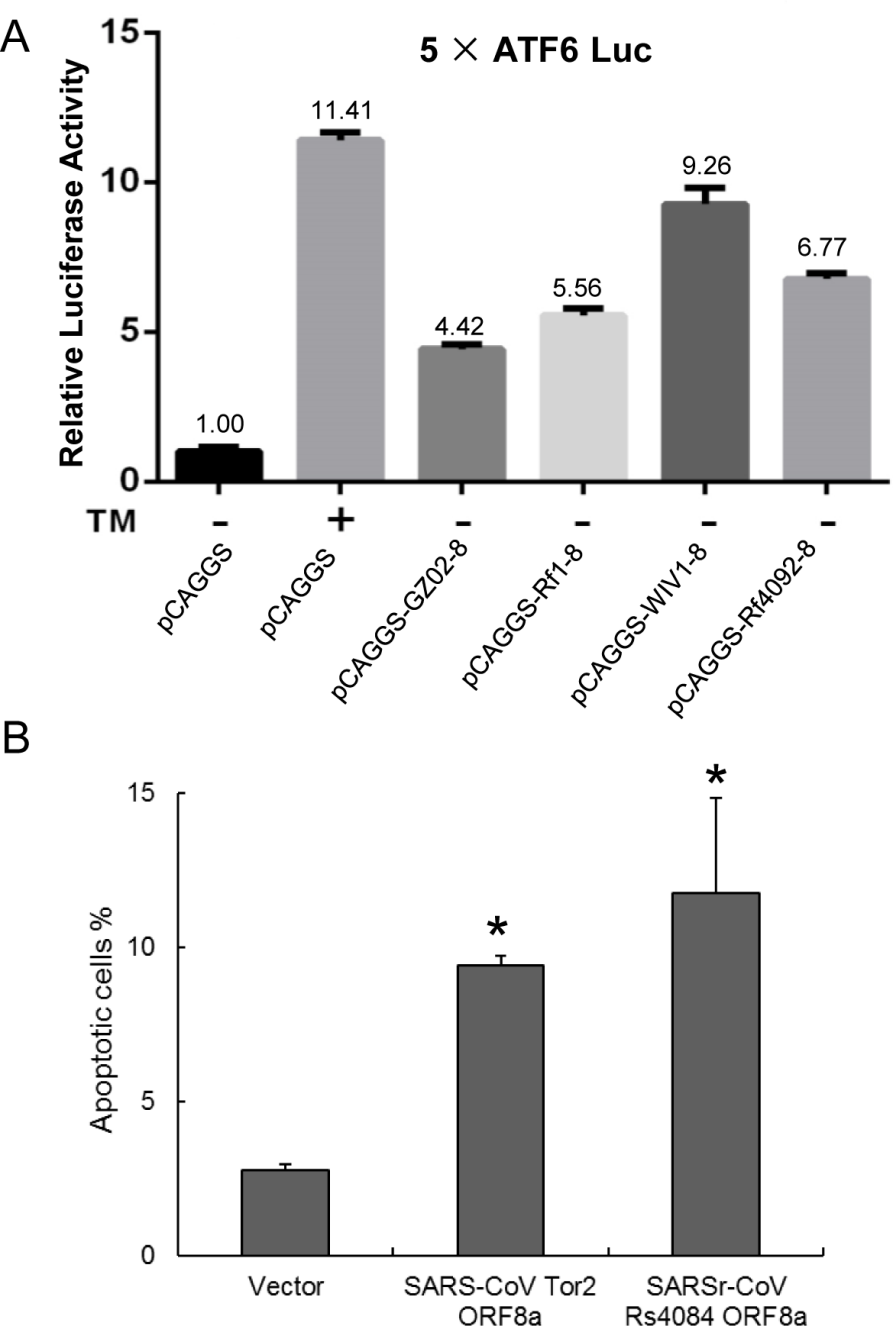
Functional characterization of diverse ORF8 and ORF8a proteins of bat SARSr-CoVs. (A) The ORF8 proteins of SARS-CoV and bat SARSr-CoVs induces the ATF6-dependent transcriptional activity. HeLa cells were transiently transfected with the pcAGGS expression plasmids of the ORF8 of SARS-CoV GZ02, bat SARSr-CoV Rf1, WIV1 and Rf4092 and the reporter plasmid 5×ATF6-GL3 for 40h. Control cells were co-transfected with the reporter plasmid and the empty pCAGGS vector for 24h, and treated with or without TM (2μg/ml) for an additional 16h. The cell lysates were harvested for dual luciferase assay and data are shown as the average values from triplicate wells. (B) The ORF8a proteins of SARS-CoV and bat SARSr-CoV triggered apoptosis. 293T cells were transfected with the expression plasmids of the ORF8a of SARS-CoV Tor2 and bat SARSr-CoV Rs4084 and a pcAGGS vector control for 24h. Apoptosis was analyzed by flow cytometry after annexin V staining and the percentage of apoptotic cells were calculated. Data are shown as the average values from triplicate cells. Error bars indicate SDs. * *P*<0.05.

The induction of the ATF6-dependent transcription by the ORF8s of SARS-CoV and bat SARSr-CoVs were investigated using a luciferase reporter, 5×ATF6-GL3. In HeLa cells transiently transfected with the expression plasmids of the ORF8s of bat SARSr-CoV Rf1, Rf4092 and WIV1, the relative luciferase activities of the 5×ATF6-GL3 reporter was enhanced by 5.56 to 9.26 folds compared with cells transfected with the pCAGGS empty vector, while it was increased by 4.42 fold by the SARS-CoV GZ02 ORF8. As a control, the treatment with tunicamyxin (TM) stimulated the transcription by about 11 folds (Fig 9A). The results suggests that various ORF8 proteins of bat SARSr-CoVs can activate ATF6, and those of some strains have a stronger effect than the SARS-CoV ORF8.

### Induction of apoptosis by the ORF8a of the newly identified bat SARSr-CoV

We conducted transient transfection to examine whether the ORF8a of SARSr-CoV Rs4084 triggered apoptosis. As shown in Fig 9B, 11.76% and 9.40% of the 293T cells transfected with the SARSr-CoV Rs4084-ORF8a and SARS-CoV Tor2-ORF8a expression plasmid underwent apoptosis, respectively. In contrast, transfection with the empty vector resulted in apoptosis in only 2.79% of the cells. The results indicate that Rs4084 ORF8a has an apoptosis induction activity similar to that of SARS-CoV [28].

## Discussion

Genetically diverse SARSr-CoVs have been detected in various horseshoe bat species across a wide geographic range in China in the past decade [9–12,14,29]. However, most bat SARSr-CoVs show considerable genetic distance to SARS-CoV, particularly in the highly variable S1, ORF8 and ORF3 regions [10,25]. Recently, several novel SARSr-CoVs have been described to be more closely related to SARS-CoV, either in the S gene or in ORF8. The S proteins of RsSHC014, Rs3367, WIV1 and WIV16, which were reported in our previous studies, shared 90% to 97% aa sequence identities to those of human/civet SARS-CoVs [17,18]. Another strain from *Rhinolophus affinis* in Yunnan termed LYRa11 showed 90% aa sequence identity to SARS-CoV in the S gene [13]. In addition, two studies have described 4 novel SARSr-CoVs (YNLF_31C/34C and GX2013/YN2013) which possessed a full-length ORF8 with substantially higher similarity to that of SARS-CoV [22,30]. These findings provide strong genetic evidence for the bat origin of SARS-CoV with regard to the S gene or ORF8. However, all of these SARSr-CoVs were distinct from SARS-CoV in at least one other gene, suggesting that none of them was the immediate progenitor of SARS-CoV. Moreover, these SARSr-CoVs were discovered in bat populations from physically distinct locations. The site of origin of the true progenitor of SARS-CoV and the evolutionary origin of SARS-CoV have until now remained elusive. In the current study, we have identified a bat habitat potentially important for SARSr-CoV evolution where a series of recombination events have likely occurred among different SARSr-CoV strains, which provides new insights into the origin of SARS-CoV.

SARS first emerged in Guangdong province in late 2002 [7]. However, SARSr-CoVs discovered in bats from neighboring areas of Guangdong to date have shown phylogenetic disparity from SARS-CoV especially in the S gene [9,10,14], suggesting SARS-CoV may have originated from another region. Our analysis of the phylogeny of SARS-CoVs and all known bat SARSr-CoVs using the nt sequence of their non-structural ORF1a and ORF1b genes, which constitute the majority of the genome, shows that SARSr-CoV evolution is strongly correlated with their geographical origin, but not host species. It is noteworthy that SARSr-CoVs detected in Yunnan are more closely related to SARS-CoV than strains from other regions in China. This finding implies that Yunnan, or southwestern China, is more likely to be the geographical source of SARS-CoV than other regions in China, but data from more extensive surveillance are yet needed to support this inference.

In our longitudinal surveillance of SARSr-CoVs in a single cave in Yunnan where we discovered Rs3367, RsSHC014, WIV1 and WIV16, the CoV prevalence in fecal samples varied among different sampling time. Generally, a higher prevalence was observed in autumn (September and October) than in spring and early summer (April and May). This may be due to the establishment of a susceptible subpopulation of newborn bats which had not developed their own immunity after the parturition period [31]. Another factor may be the changes in the composition of bat species in the cave at different sampling dates. For example, in September 2012 when the CoV prevalence reached 51.3%, the majority of samples were from *R*. *sinicus*, but in May 2015 when only 3 out of the 145 samples tested positive, *Aselliscus stoliczkanus* was the predominant bat species in the cave. We failed to amplify the RBD sequences from 15 of the 64 SARSr-CoV positive samples. Most of these samples had comparatively low viral concentration (< 10^7^ copies/g) (S8 Fig), as revealed by our previous quantitative studies [32]. The unsuccessful amplification of RBD in some samples with high viral concentration was probably because of the more divergent sequences in this region of these SARSr-CoV genomes.

In this cave, we have now obtained full-length genome sequences of additional 11 novel SARSr-CoVs from bats. Our findings suggest the co-circulation of different bat SARSr-CoVs highly similar to SARS-CoV in the most variable S1 (NTD and RBD), ORF8 and ORF3 regions, respectively, in this single location. In the ORF1a, ORF1b, E, M and N genes, the SARSr-CoVs circulating in this cave also shared > 98% aa sequence identities with human/civet SARS-CoVs. Thus, all of the building blocks of the SARS-CoV genome were present in SARSr-CoVs from this single location in Yunnan during our sampling period. Furthermore, strains closely related to different representative bat SARSr-CoVs from other provinces (e.g. Rs672, HKU3 and Rf1) in the RBD region were also detected there. Therefore, this cave could be regarded as a rich gene pool of bat SARSr-CoVs, wherein concurrent circulation of a high diversity of SARSr-CoV strains has led to an unusually diverse assemblage of SARSr-CoVs.

During our 5-year surveillance in this single cave, we first reported Rs3367 and WIV1 in 2013, with RBD sequence closely resembling that of SARS-CoV [17]. More recently, we discovered WIV16 which had an RBD almost identical to WIV1’s but shared much higher similarity with SARS-CoV than WIV1 in the NTD region of S1, making it the closest SARSr-CoV to the epidemic strains identified to date [18]. In this study, we found a novel strain Rs4231 from the same location sharing almost identical NTD sequence with WIV16 but distinct from it in the RBD, with evidence of a recombination event. Our recombination analysis indicated that a recombination event may have taken place at the junction between the coding region of NTD and RBD in the Rs4231 and WIV1 genomes and resulted in WIV16. Recombination at this genomic position also happened among other SARSr-CoVs relatively distant to SARS-CoV found in this location (e.g. Rs4081 and Rs4247, S5 Fig). The frequent recombination at this hotspot in the S gene increased the genetic diversity of SARSr-CoVs harbored in these bat populations and might have been responsible for the generation of the S gene of the direct progenitor strain of SARS-CoV.

The genomes of SARS-CoVs from patients during the early epidemic phase and civet SARS-CoVs all contained a single full-length ORF8 [3,7]. We have found that a number of bat SARSr-CoVs from this cave possessed a complete ORF8 highly similar to that of early human/civet SARS-CoV (>97% nt sequence identity), represented by strain Rf4092 (S3C Fig). This provided further evidence for the source of human SARS-CoV ORF8 in bats [22,30]. In contrast, the ORF8 was split into overlapping ORF8a and ORF8b in most human SARS-CoV strains from later-phase patients due to the acquisition of a 29-nt deletion [8,26]. In this study, we have discovered for the first time a bat SARSr-CoV with ORF8a and ORF8b highly similar to the later-phase human SARS-CoVs, though the split of ORF8 in the bat SARSr-CoV and that in human SARS-CoV were two independent events. Our recombination analysis suggests that this strain, Rs4084, likely acquired its ORF8 from Rf4092 through recombination, followed by the development of the 5-nt deletion which led to the splitting. It suggests that ORF8 region in bat SARSr-CoV genomes is prone to deletions as in human SARS-CoV [3,25]. Finally, the recombination analysis suggests that an ancestral strain of SARS-CoV SZ3 would have been generated if the recombination around ORF8 had occurred between the lineages that led to WIV16 and Rf4092. Taken together, the evidence of recombination events among SARSr-CoVs harbored by bats in this single location suggests that the direct progenitor of SARS-CoV may have originated as a result of a series of recombination within the S gene and around ORF8. This could have been followed by the spillover from bats to civets and people either in the region, or during movement of infected animals through the wildlife trade. However, given the paucity of data on animal trade prior to the SARS outbreak, the likely high geographical sampling bias in bat surveillance for SARSr-CoVs in southern China, and the possibility that other caves harbor similar bat species assemblages and a rich diversity of SARSr-CoVs, a definite conclusion about the geographical origin of SARS-CoV cannot be drawn at this point.

*R*. *sinicus* are regarded as the primary natural host of SARS-CoV, as all SARSr-CoVs highly homologous to SARS-CoV in the S gene were predominantly found in this species. However, it is noted that two SARSr-CoVs previously reported from *R*. *ferrumequinum* showed the closest phylogenetic position to SARS-CoV in the ORF1a/1b trees. These strains were discovered in another location in Yunnan 80 km from the cave surveyed in the current study [22]. This information also supports the speculation that SARS-CoV may have originated from this region. Nonetheless, since the correlation between the host species and the phylogeny of SARSr-CoV ORF1ab seems limited, more SARSr-CoV sequences need to be obtained from different *Rhinolophus* bat species in both locations in Yunnan, and from other locations in southern China. In particular, it will be important to assess whether *R*. *ferrumequinum* played a more important role in the evolution of SARS-CoV ORF1ab.

The cave we studied is located approximately 60 km from the city of Kunming. Beside a number of rhinolophid and hipposiderid species from which SARSr-CoVs have been detected, other bats like myotis were also present there. The temperature in the cave is around 22–25°C and the humidity around 85%-90%. The physical nature of the cave is not unique, but it does appear to host a particularly dense population of bats in the reproductive season. Similar caves co-inhabited by bat populations of different species are not rare in other areas in Yunnan. We propose that efforts to study the ecology, host species diversity, and viral strain populations of these caves may provide critical information on what drives SARSr-CoV evolution.

Our previous studies demonstrated the capacity of both WIV1 and WIV16 to use ACE2 orthologs for cell entry and to efficiently replicate in human cells [17,18]. In this study, we confirmed the use of human ACE2 as receptor of two novel SARSr-CoVs by using chimeric viruses with the WIV1 backbone replaced with the S gene of the newly identified SARSr-CoVs. Rs7327’s S protein varied from that of WIV1 and WIV16 at three aa residues in the receptor-binding motif, including one contact residue (aa 484) with human ACE2. This difference did not seem to affect its entry and replication efficiency in human ACE2-expressing cells. A previous study using the SARS-CoV infectious clone showed that the RsSHC014 S protein could efficiently utilize human ACE2 [33], despite being distinct from SARS-CoV and WIV1 in the RBD (S1 Fig). We examined the infectivity of Rs4231, which shared similar RBD sequence with RsSHC014 but had a distinct NTD sequence, and found the chimeric virus WIV1-Rs4231S also readily replicated in HeLa cells expressing human ACE2 molecule. The novel live SARSr-CoV we isolated in the current study (Rs4874) has an S gene almost identical to that of WIV16. As expected, it is also capable of utilizing human ACE2. These results indicate that diverse variants of SARSr-CoV S protein without deletions in their RBD are able to use human ACE2. In contrast, our previous study revealed that the S protein of a *R*. *sinicus* SARSr-CoV with deletions (Rp3) failed to use human, civet and bat ACE2 for cell entry [34]. In this study, in addition to Rs4231 and Rs7327, we also constructed infectious clones with the S gene of Rs4081, Rf4075, Rs4085, Rs4235 and As6526, which all contained the deletions in their RBD. These 7 strains, plus Rs4874 and the previously studied WIV1 and RsSHC014, could represent all types of S variants of SARSr-CoVs in this location (S3A Fig). However, none of the strains with deletions in the RBD could be rescued from Vero E6 cells. Therefore, the two distinct clades of SARSr-CoV S gene may represent the usage of different receptors in their bat hosts.

The full-length ORF8 protein of SARS-CoV is a luminal endoplasmic reticulum (ER) membrane-associated protein that induces the activation of ATF6, an ER stress-regulated transcription factor that activates the transcription of ER chaperones involved in protein folding [35]. We amplified the ORF8 genes of Rf1, Rf4092 and WIV1, which represent three different genotypes of bat SARSr-CoV ORF8 (S3C Fig), and constructed the expression plasmids. All of the three ORF8 proteins transiently expressed in HeLa cells can stimulate the ATF6-dependent transcription. Among them, the WIV1 ORF8, which is highly divergent from the SARS-CoV ORF8, exhibited the strongest activation. The results indicate that the variants of bat SARSr-CoV ORF8 proteins may play a role in modulating ER stress by activating the ATF6 pathway. In addition, the ORF8a protein of SARS-CoV from the later phase has been demonstrated to induce apoptosis [28]. In this study, we have found that the ORF8a protein of the newly identified SARSr-CoV Rs4084, which contained an 8-aa insertion compared with the SARS-CoV ORF8a, significantly triggered apoptosis in 293T cells as well.

Compared with the 154-aa ORF3b of SARS-CoV, the ORF3b proteins of all previously identified bat SARSr-CoVs were smaller in size due to the early translation termination. However, for the first time, we discovered an ORF3b without the C-terminal truncation in a bat SARSr-CoV, Rs7327, which differed from the ORF 3b of SARS-CoV GZ02 strain at only one aa residue. The SARS-CoV ORF3b antagonizes interferon function by modulating the activity of IFN regulatory factor 3 (IRF3) [27]. As previous studies suggested, the nuclear localization signal-containing C-terminal may not be required for the IFN antagonist activity of ORF3b [36]. Our previous studies also demonstrated that the ORF3b protein of a bat SARSr-CoV, termed Rm1, which was C-terminally truncated to 56 aa and shared 62% aa sequence identity with SARS-CoV, still displayed the IFN antagonist activity [37]. It is very interesting to investigate in further studies whether Rs7327’s ORF3b and other versions of truncated ORF3b such as WIV1 and WIV16 also show IFN antagonism profiles.

As a whole, our findings from a 5-year longitudinal study conclusively demonstrate that all building blocks of the pandemic SARS-CoV genome are present in bat SARSr-CoVs from a single location in Yunnan. The data show that frequent recombination events have happened among those SARSr-CoVs in the same cave. While we cannot rule out the possibility that similar gene pools of SARSr-CoVs exist elsewhere, we have provided sufficient evidence to conclude that SARS-CoV most likely originated from horseshoe bats via recombination events among existing SARSr-CoVs. In addition, we have also revealed that various SARSr-CoVs capable of using human ACE2 are still circulating among bats in this region. Thus, the risk of spillover into people and emergence of a disease similar to SARS is possible. This is particularly important given that the nearest village to the bat cave we surveyed is only 1.1 km away, which indicates a potential risk of exposure to bats for the local residents. Thus, we propose that monitoring of SARSr-CoV evolution at this and other sites should continue, as well as examination of human behavioral risk for infection and serological surveys of people, to determine if spillover is already occurring at these sites and to design intervention strategies to avoid future disease emergence.

## Materials and methods

### Ethics statement

All sampling procedures were performed by veterinarians with approval from Animal Ethics Committee of the Wuhan Institute of Virology (WIVH05210201). The study was conducted in accordance with the Guide for the Care and Use of Wild Mammals in Research of the People’s Republic of China.

### Sampling

Bat samplings were conducted ten times from April 2011 to October 2015 at different seasons in their natural habitat at a single location (cave) in Kunming, Yunnan Province, China. All members of field teams wore appropriate personal protective equipment, including N95 masks, tear-resistant gloves, disposable outerwear, and safety glasses. Bats were trapped and fecal swab samples were collected as described previously [9]. Clean plastic sheets measuring 2.0 by 2.0 m were placed under known bat roosting sites at about 18:00 h each evening for collection of fecal samples. Fresh fecal pellets were collected from sheets early in the next morning. Each sample (approximately 1 gram of fecal pellet) was collected in 1ml of viral transport medium composed of Hank's balanced salt solution at pH7.4 containing BSA (1%), amphotericin (15 μg/ml), penicillin G (100 units/ml), and streptomycin (50 μg/ml), and were stored at -80°C until processing. Bats trapped for this study were released back into their habitat.

### RNA extraction, PCR screening and sequencing

Fecal swab or pellet samples were vortexed for 1 min, and 140 μl of supernatant was collected from each sample after centrifuge at 3000 rpm under 4°C for 1min. Viral RNA was extracted with Viral RNA Mini Kit (Qiagen) following the manufacturer’s instructions. RNA was eluted in 60 μl of buffer AVE (RNase-free water with 0.04% sodium azide, Qiagen), aliquoted, and stored at -80°C. One-step hemi-nested RT-PCR (Invitrogen) was employed to detect the presence of coronavirus sequences as described previously using a set of primers that target a 440-nt fragment in the RNA-dependent RNA polymerase gene (*RdRp*) of all known alpha- and betacoronaviruses [20]. For the first round PCR, the 25 μl reaction mix contained 12.5 μl PCR 2 × reaction mix buffer, 10 pmol of each primer, 2.5 mM MgSO_4_, 20 U RNase inhibitor, 1 μl SuperScript III/Platinum Taq Enzyme Mix and 5 μl RNA template. The amplification was performed as follows: 50°C for 30 min, 94°C for 2 min, followed by 40 cycles consisting of 94°C for 15 sec, 52°C for 30 sec, 68°C for 40 sec, and a final extension of 68°C for 5 min. For the second round PCR, the 25 μl reaction mix contained 2.5 μl PCR reaction buffer, 5 pmol of each primer, 50 mM MgCl_2_, 0.5mM dNTP, 0.1 μl Platinum Taq Enzyme (Invitrogen) and 1 μl product of the first round PCR. The amplification was performed as follows: 94°C for 3 min followed by 35 cycles consisting of 94°C for 30 sec, 52°C for 30 sec, 72°C for 40 sec, and a final extension of 72°C for 7 min. The RBD region was amplified using the one-step nested RT-PCR method previously described [17].

PCR products were gel purified and sequenced with an ABI Prism 3730 DNA analyzer (Applied Biosystems, USA). PCR products with low concentration or generating heterogeneity in the sequencing chromatograms were cloned into pGEM-T Easy Vector (Promega) for sequencing. The positive samples in this study were termed using the abbreviated name of bat species plus the sample ID number (e.g. Rs4081). To confirm the bat species of individual sample, PCR amplification of cytochrome b (*Cytob*) or NADH dehydrogenase subunit 1 (*ND1*) gene was performed using DNA extracted from the feces or swabs [38,39].

### Sequencing of full-length genomes

Full genomic sequences of 11 SARSr-CoVs were determined by One-step PCR (Invitrogen) amplification of overlapping genomic fragments with degenerate primers designed by multiple alignment of available SARS-CoV and bat SARSr- CoV sequences deposited in GenBank, and additional specific primers designed from the results of previous rounds of sequencing in this study. Primer sequences are available upon request. Sequences of the 5’ and 3’ genomic ends were obtained by 5’ and 3’ RACE (Roche), respectively. PCR products with expected size were gel-purified and subjected directly to sequencing. Each fragment was sequenced at least twice. The sequencing chromatogram of each product was thoroughly examined and sequence heterogeneity was not observed. For some fragments with low concentration of amplicons, the PCR products were cloned into pGEM-T Easy Vector (Promega) for sequencing. At least five independent clones were sequenced to obtain a consensus sequence. Co-presence of sequences of distinct SARSr-CoVs was not found in any of the amplicons. The sequences of overlapping genomic fragments were assembled to obtain the full-length genome sequences, with each overlapping sequence longer than 100 bp.

### Evolution analysis

Full-length genome sequences of the 15 SARSr-CoVs detected from bats in the cave surveyed in this study were aligned with those of selected SARS-CoVs using MUSCLE [40]. The aligned sequences were scanned for recombination events by Recombination Detection Program (RDP) [41]. The potential recombination events suggested by strong *P* values (<10^−20^) were further confirmed using similarity plot and bootscan analyses implemented in Simplot 3.5.1 [42]. Phylogenetic trees based on nucleotide sequences were constructed using the Maximum Likelihood algorithm under the LG model with bootstrap values determined by 1000 replicates in the PhyML (version 3.0) software package [43].

### Virus isolation

The Vero E6 cell line was kindly provided by Australian Animal Health Laboratory, CSIRO (Geelong, Australia). Vero E6 monolayer was maintained in DMEM medium supplemented with 10% fetal calf serum (FCS). Fecal samples (in 200 μl buffer) were gradient centrifuged at 3,000–12,000 g, and the supernatant was diluted 1:10 in DMEM before being added to Vero E6 cells. After incubation at 37°C for 1 h, the inoculum was removed and replaced with fresh DMEM medium with 2% FCS. The cells were incubated at 37°C and checked daily for cytopathic effect. All tissue culture media were supplemented with triple antibiotics penicillin/ streptomycin/amphotericin (Gibco) (penicillin 200 IU/ml, streptomycin 0.2 mg/ml, amphotericin 0.5 μg/ml). Three blind passages were carried out for each sample. After each passage, both the culture supernatant and cell pellet were examined for presence of SARSr-CoV by RT-PCR using specific primers targeting the RdRp or S gene. The viruses which caused obvious cytopathic effect and could be detected in three blind passages by RT-PCR were further confirmed by electron microscopy.

### Construction of recombinant viruses

Recombinant viruses with the S gene of the novel bat SARSr-CoVs and the backbone of the infectious clone of SARSr-CoV WIV1 were constructed using the reverse genetic system described previously [23] (S9 Fig). The fragments E and F were re-amplified with primer pairs (FE, 5’-AGGGCCCACCTGGCACTGGTAAGAGTCATTTTGC-3’, R-EsBsaI, 5’-ACTGGTCTCTTCGTTTAGTTATTAACTAAAATATCACTAGACACC-3’) and (F-FsBsaI, 5’-TGAGGTCTCCGAACTTATGGATTTGTTTATGAG-3’, RF, 5’-AGGTAGGCCTCTAGGGCAGCTAAC-3’), respectively. The products were named as fragment Es and Fs, which leave the spike gene coding region as an independent fragment. BsaI sites (5’-GGTCTCN|NNNN-3’) were introduced into the 3’ terminal of the Es fragment and the 5’ terminal of the Fs fragment, respectively. The spike sequence of Rs4231 was amplified with the primer pair (F-Rs4231-BsmBI, 5’-AGTCGTCTCAACGAACATGTTTATTTTCTTATTCTTTCTCACTCTCAC-3’ and R-Rs4231-BsmBI, 5’-TCACGTCTCAGTTCGTTTATGTGTAATGTAATTTGACACCCTTG-3’). The S gene sequence of Rs7327 was amplified with primer pair (F-Rs7327-BsaI, 5’-AGTGGTCTCAACGAACATGAAATTGTTAGTTTTAGTTTTTGCTAC-3’ and R-Rs7327-BsaI, 5’- TCAGGTCTCAGTTCGTTTATGTGTAATGTAATTTAACACCCTTG-3’). The fragment Es and Fs were both digested with BglI (NEB) and BsaI (NEB). The Rs4231 S gene was digested with BsmBI. The Rs7327 S gene was digested with BsaI. The other fragments and bacterial artificial chromosome (BAC) were prepared as described previously. Then the two prepared spike DNA fragments were separately inserted into BAC with Es, Fs and other fragments. The correct infectious BAC clones were screened. The chimeric viruses were rescued as described previously [23].

### Determination of virus infectivity by immunofluorescence assay

The HeLa cell line was kindly provided by Australian Animal Health Laboratory, CSIRO (Geelong, Australia). HeLa cells expressing human ACE2 were constructed as described previously [17]. HeLa cells expressing human ACE2 and Vero E6 cells were cultured on coverslips in 24-well plates (Corning) incubated with the newly isolated or recombinant bat SARSr-CoVs at a multiplicity of infection (MOI) = 1.0 for 1h. The inoculum was removed and the cells were washed twice with PBS and supplemented with medium. Vero E6 cells without virus inoculation and HeLa cells without ACE2 were used as negative control. Twenty-four hours after infection, cells were rinsed with PBS and fixed with 4% formaldehyde in PBS (pH7.4) at 4°C for 20 min. ACE2 expression was detected by using goat anti-human ACE2 immunoglobulin followed by FITC-labelled donkey anti-goat immunoglobulin (PTGLab). Virus replication was detected by using rabbit antibody against the nucleocapsid protein of bat SARSr-CoV Rp3 followed by Cy3-conjugated mouse anti-rabbit IgG. Nuclei were stained with DAPI. Staining patterns were observed under an FV1200 confocal microscope (Olympus).

### Determination of virus replication in Vero E6 cells by plaque assay

Vero E6 cells were infected with WIV1, Rs4874, WIV1-Rs4231S, and WIV1-Rs7327S at an MOI of 1.0 and 0.01. After incubation for an hour, the cells were washed with DHanks for three times and supplied with DMEM containing 2% FCS. Samples were collected at 0, 10, 27, and 48 h post infection. The viral titers were determined by plaque assay.

### Determination of virus replication in HeLa cells expressing human ACE2 by quantitative RT-PCR

HeLa cells expressing human ACE2 were inoculated with WIV1, Rs4874, WIV1-Rs4231S, and WIV1-Rs7327S at an MOI of 1.0, and were incubated for 1h at 37°C. After the inoculum was removed, the cells were supplemented with medium containing 1% FBS. Supernatants were collected at 0, 12, 24 and 48h. Virus titers were determined using quantitative RT-PCR targeting the partial N gene with a standard curve which expresses the correlation between Ct value and virus titer (shown as TCID50/ml). The standard curve was made using RNA dilutions from the purified Rs4874 virus stock (with a titer of 2.15 × 10^6^ TCID50/ml). For qPCR, RNA was extracted from 140 μl of each supernatant with Viral RNA Mini Kit (Qiagen) following manufacturer’s instructions and eluted in 60 μl AVE buffer. The PCR was performed with the TaqMan AgPath-ID One-Step RT–PCR Kit (Applied Biosystems) in a 25 μl reaction mix containing 4 μl RNA, 1 × RT–PCR enzyme mix, 1 × RT–PCR buffer, 40 pmol forward primer (5’-GTGGTGGTGACGGCA AAATG-3’), 40 pmol reverse primer (5’-AAGTGAAGCTTCTGGGCCAG-3’) and 12 pmol probe (5’-FAM-AAAGAGCTCAGCCCCAGATG-BHQ1-3’). The amplification was performed as follows: 50°C for 10 min, 95°C for 10 min followed by 50 cycles consisting of 95°C for 15 sec and 60°C for 20 sec.

### Plasmids

The ORF8 genes of bat SARSr-CoV WIV1 and Rf4092 and the ORF8a gene of bat SARSr-CoV Rs4084 were amplified by PCR from the viral RNA extracted from the isolated virus or fecal samples. The ORF8 gene of SARS-CoV GZ02 and bat SARSr-CoV Rf1, and the ORF8a gene of SARS-CoV Tor2 were synthesized by Tsingke Biological Technology Co., Ltd (Wuhan, China). All genes were cloned into the pCAGGS vector constructed with a C-terminal HA tag. Expression of the proteins was confirmed by Western blotting using a mAb against the HA tag. Five tandem copies of the ATF6 consensus binding sites were synthesized and inserted into the pGL3-Basic vector to construct the luciferase reporter plasmid 5×ATF6-GL3, in which the luciferase gene is under the control of the c-*fos* minimal promoter and the ATF6 consensus binding sites.

### Luciferase reporter assay

HeLa cells in 24-well plates were transfected using Lipofectamine 3000 reagent (Life Technologies) following the manufacturer’s instruction. Cells per well were co-transfected with 600ng of the 5×ATF6-GL3 reporter plasmid, with 300ng of each expression plasmid of SARS-CoV and SARSr-CoV ORF8 or empty vector and 20ng of pRL-TK (Promega) which served as an internal control. The cells were incubated for 24h, and were treated with or without 2μg/ml tunicamycin for 16h. Cells were harvested and lysed. Luciferase activity was determined using a dual-luciferase assay system (Promega). The experiment was performed in triplicate wells.

### Quantification of apoptotic cells

293T cells in 12-well plates were transfected using Lipofectamine 3000 reagent (Life Technologies) following the manufacturer’s instruction. Cells per well were transfected with 3μg of the expression plasmid of SARS-CoV Tor2 or SARSr-CoV Rs4084 ORF8a, or the empty vector. 24h post transfection, apoptotic cells were quantified by using the Annexin V-fluorescein isothiocyanate (FITC)/PI Apoptosis Detection Kit (Yeasen Biotech, Shanghai) in accordance with the manufacturer’s instruction. Apoptosis was analyzed by flow cytometry. The experiment was performed in triplicate wells.

### Accession numbers

The complete genome sequences of bat SARS-related coronavirus strains As6526, Rs4081, Rs4084, Rf4092, Rs4231, Rs4237, Rs4247, Rs4255, Rs4874, Rs7327 and Rs9401 have been deposited in the GenBank database with the accession numbers from KY417142 to KY417152, respectively.

## Supporting information

S1 Fig Alignment of amino acid sequences of the receptor-binding motif (corresponding to aa 424–495 of SARS-CoV S protein). Two clades of the SARSr-CoVs identified from bats in the studied cave are indicated with vertical lines on the left.
(PPTX)

S2 Fig Alignment of nucleotide sequences of a genomic region covering ORF6 to ORF7a. ORFX is located between ORF6 and ORF7a in the genomes of WIV1, WIV16, Rs7327 and Rs4874. The start codon and stop codon of ORFX are marked with red boxes. The deletion responsible for the long ORFX in Rs7327 and Rs4874 is marked with the blue box.
(PPTX)

S3 Fig **Phylogenetic analyses based on nucleotide sequences of the S gene (A), ORF3a (B) and ORF8 (C).** The trees were constructed by the maximum likelihood method using the LG model with bootstrap values determined by 1000 replicates. Only bootstraps > 50% are shown. Rs, *Rhinolophus sinicus*; Rf, *Rhinolophus ferremequinum*; Rm, *Rhinolophus macrotis*; Ra, *Rhinolophus affinis*; Rp, *Rhinolophus pusillus*; As, *Aselliscus stoliczkanus*; Cp, *Chaerephon plicata*. SARSr-CoVs detected in bats from the single cave surveyed in this study are in bold.
(PPTX)

S4 Fig Alignment of amino acid sequences of ORF3b protein. (PPTX)

S5 Fig Detection of potential recombination events by similarity plot and boot scan analysis. (A) Full-length genome sequence of SARSr-CoV Rs4084 was used as query sequence and RsSHC014, Rf4092 and Rs4081 as reference sequences. (B) Full-length genome sequence of SARSr-CoV Rs4237 was used as query sequence and SARSr-CoV Rs4247, Rs4081 and Rs3367 as reference sequences. All analyses were performed with a Kimura model, a window size of 1500 base pairs, and a step size of 150 base pairs.
(PPTX)

S6 Fig Chinese provinces where bat SARSr-CoVs have been detected. (PPTX)

S7 Fig The successful or failed rescue of the chimeric SARSr-CoVs. (A) Cytopathic effects in Vero E6 cells transfected with the infectious BAC clones constructed with the backbone of WIV1 and various S genes of different bat SARSr-CoV strains. Microphotographs were taken 24 hours post transfection. (B) The culture media supernatant collected from the cells transfected with the infectious BAC clones was used to infect Vero E6 cells. Immunofluorescent assay (IFA) was performed to detect infection and viral replication. Cells were fixed 24 hours post infection, and stained using rabbit antibody against the SARSr-CoV Rp3 nucleocapsid protein and a Cy3-conjugated anti-rabbit IgG.
(PPTX)

S8 Fig Quantification of SARSr-CoV in individual bat fecal samples. The number of genome copies of SARSr-CoV per gram of bat feces was determined by quantitative real-time PCR targeting the RdRp gene. Samples from which the SARSr-CoV RBD sequences were successfully amplified are indicated in red.
(PPTX)

S9 Fig Spike substitution strategy. The original fragments E and F were shortened to leave spike gene as an independent fragment. The new fragments were designated as Es and Fs. BsaI or BsmBI sites were introduced into the junctions of Es/Spike and Spike/Fs. Then any spike could be substituted into the genome of SARSr-CoV WIV1 through this strategy.
(TIF)

S1 Table Comparison of the novel bat SARSr-CoVs identified in this study with human/civet SARS-CoVs and previously described bat SARSr-CoVs. (DOCX)

S2 Table Distribution of SARSr-CoVs highly similar to SARS-CoV in the variable S, ORF3 and ORF8 genes in the single cave. (DOCX)

S1 Dataset Full-length genome sequences of bat SARSr-CoVs newly identified in this study. (FAS)
