# Supplementary figures and images for: Discovery of a rich gene pool of bat SARS-related coronaviruses provides new insights into the origin of SARS coronavirus
Source: PLoS Pathog. 2017 Nov 30;13(11):e1006698. doi: 10.1371/journal.ppat.1006698 (PMC5708621; doi:10.1371/journal.ppat.1006698)

## Slide 1
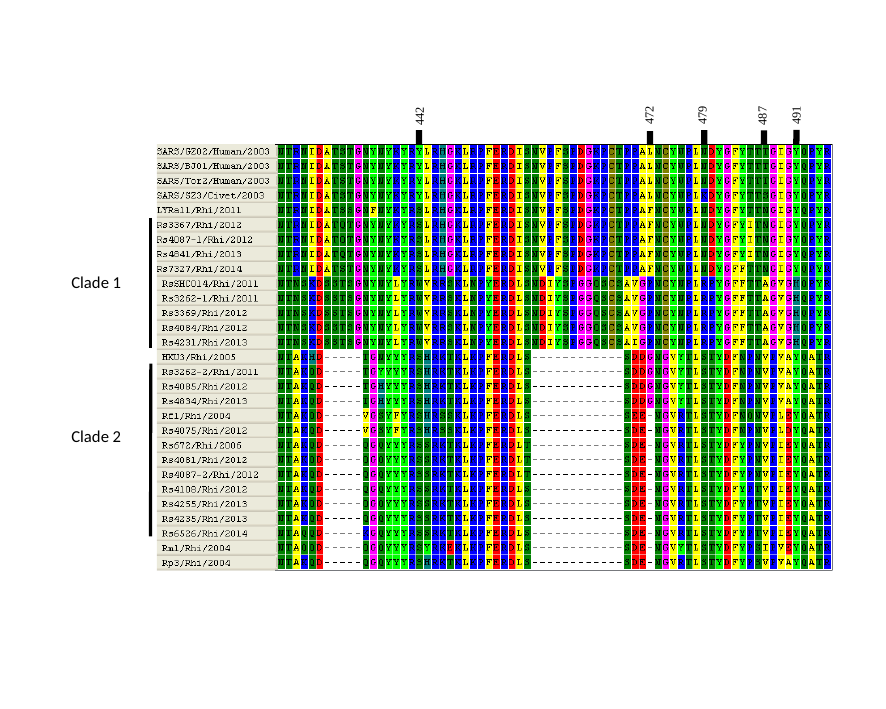

479
 491
 472
 487
 442
Clade 1
Clade 2

Supplement: S1 Fig — Two clades of the SARSr-CoVs identified from bats in the studied cave are indicated with vertical lines on the left. (PPTX) [file ppat.1006698.s001.pptx]

## Slide 1
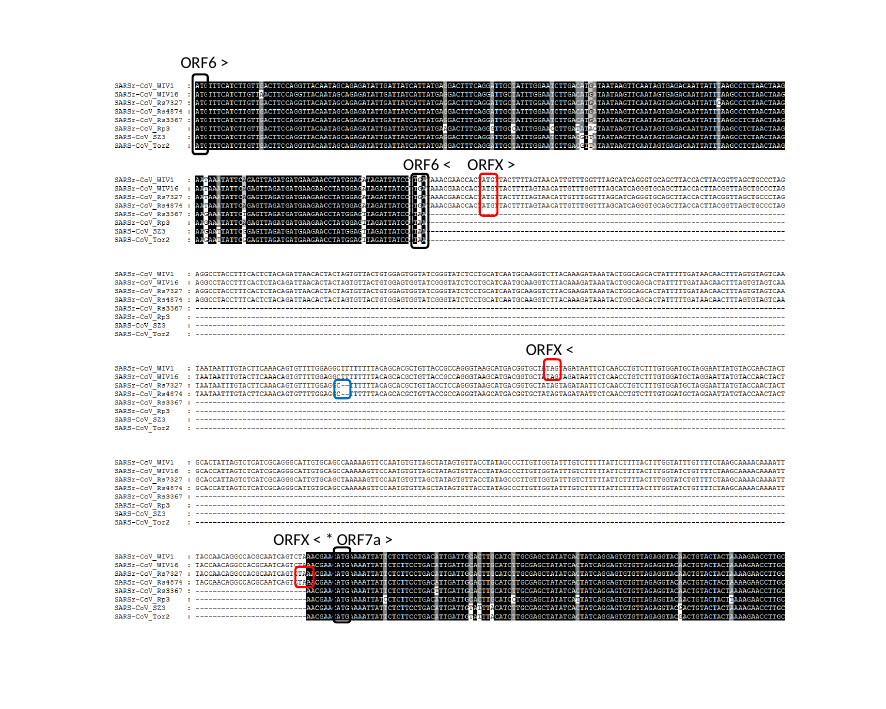

ORF6 >
ORF6 <
ORFX >
ORFX <
ORFX < *
ORF7a >

Supplement: S2 Fig — ORFX is located between ORF6 and ORF7a in the genomes of WIV1, WIV16, Rs7327 and Rs4874. The start codon and stop codon of ORFX are marked with red boxes. The deletion responsible for the long ORFX in Rs7327 and Rs4874 is marked with the blue box. (PPTX) [file ppat.1006698.s002.pptx]

## Slide 1
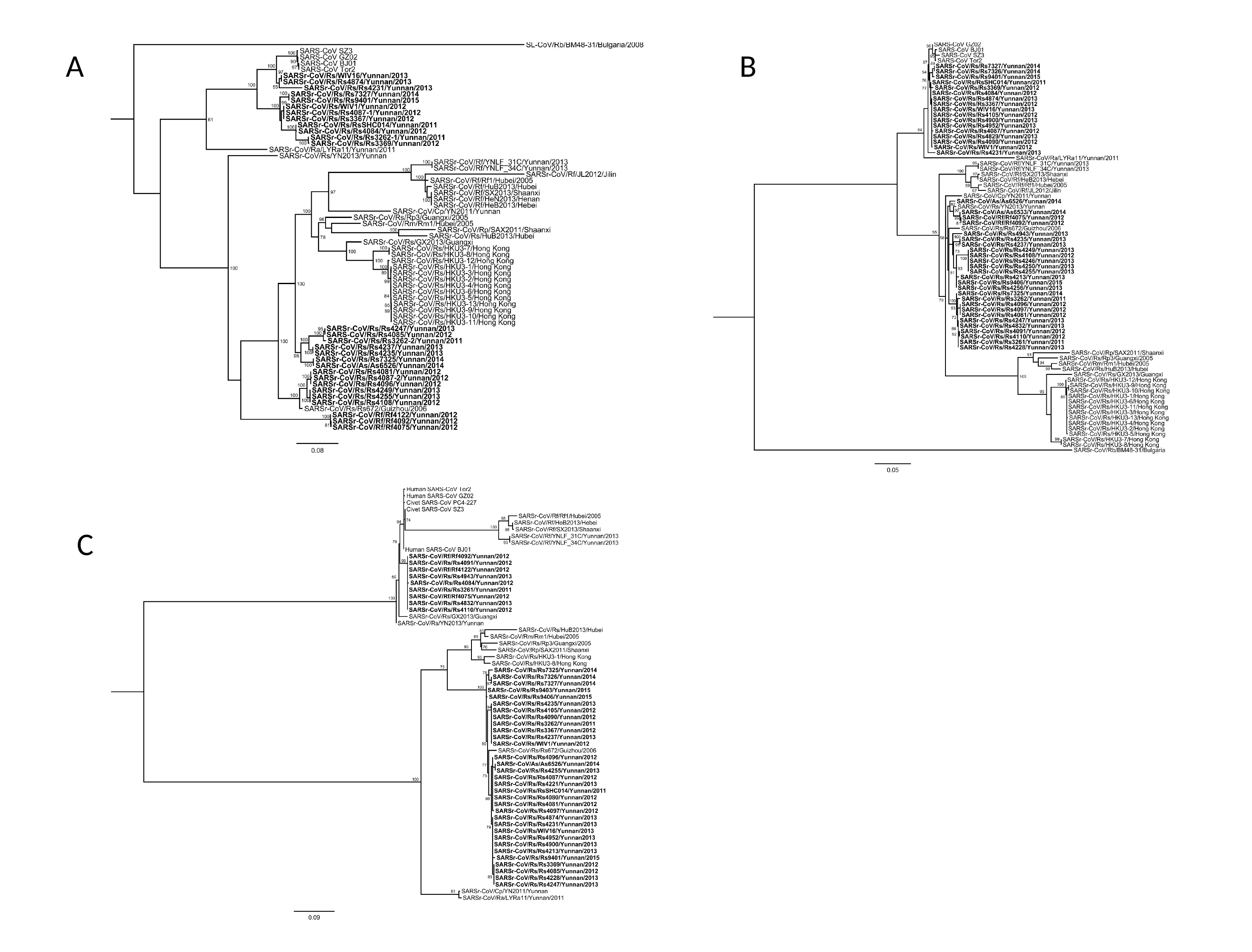

B
A
C

Supplement: S3 Fig — Phylogenetic analyses based on nucleotide sequences of the S gene (A), ORF3a (B) and ORF8 (C). The trees were constructed by the maximum likelihood method using the LG model with bootstrap values determined by 1000 replicates. Only bootstraps > 50% are shown. Rs, Rhinolophus sinicus; Rf, Rhinolophus ferremequinum; Rm, Rhinolophus macrotis; Ra, Rhinolophus affinis; Rp, Rhinolophus pusillus; As, Aselliscus stoliczkanus; Cp, Chaerephon plicata. SARSr-CoVs detected in bats from the single cave surveyed in this study are in bold. (PPTX) [file ppat.1006698.s003.pptx]

## Slide 1
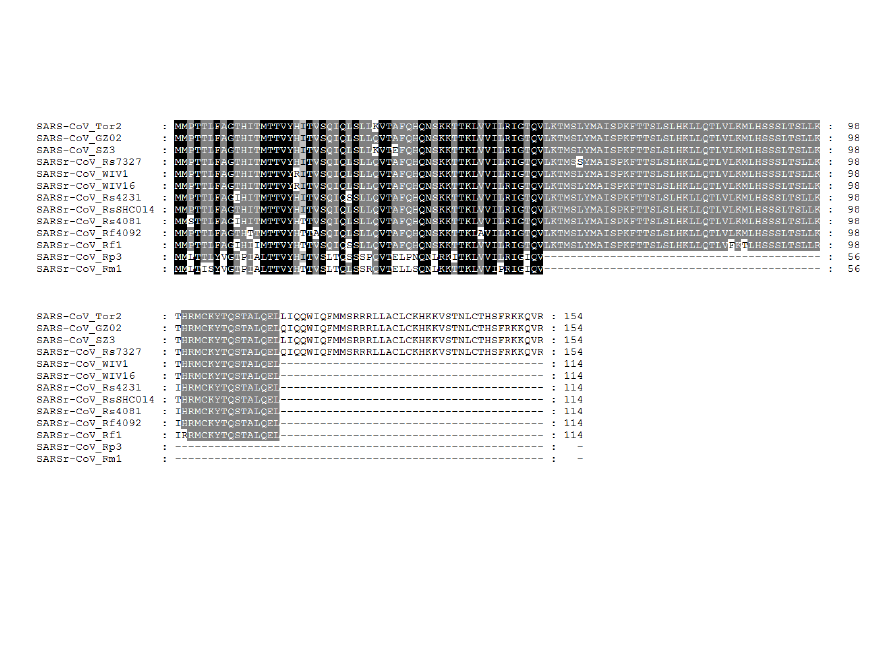

Supplement: S4 Fig — (PPTX) [file ppat.1006698.s004.pptx]

## Slide 1
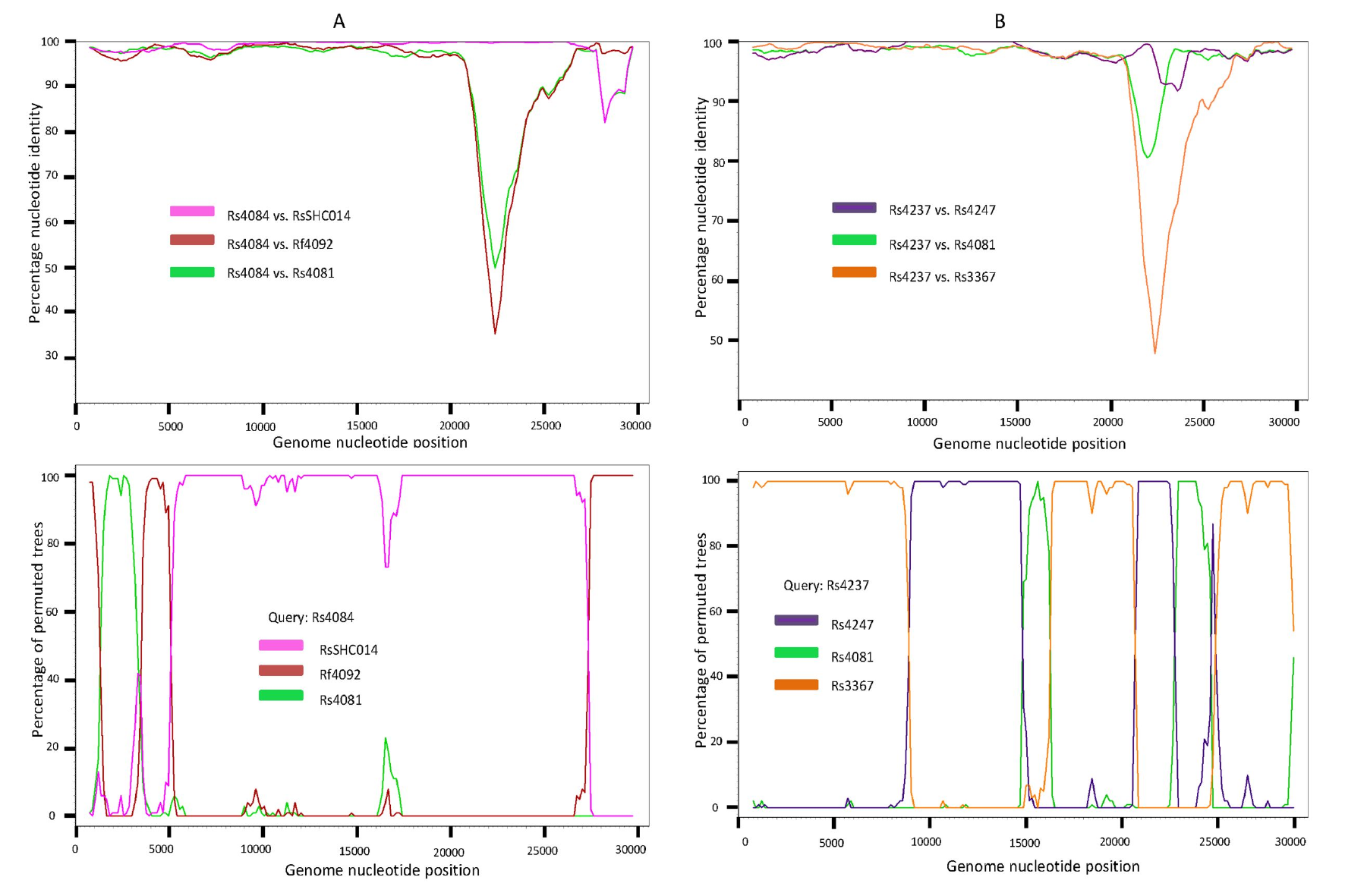

Supplement: S5 Fig — (A) Full-length genome sequence of SARSr-CoV Rs4084 was used as query sequence and RsSHC014, Rf4092 and Rs4081 as reference sequences. (B) Full-length genome sequence of SARSr-CoV Rs4237 was used as query sequence and SARSr-CoV Rs4247, Rs4081 and Rs3367 as reference sequences. All analyses were performed with a Kimura model, a window size of 1500 base pairs, and a step size of 150 base pairs. (PPTX) [file ppat.1006698.s005.pptx]

## Slide 1
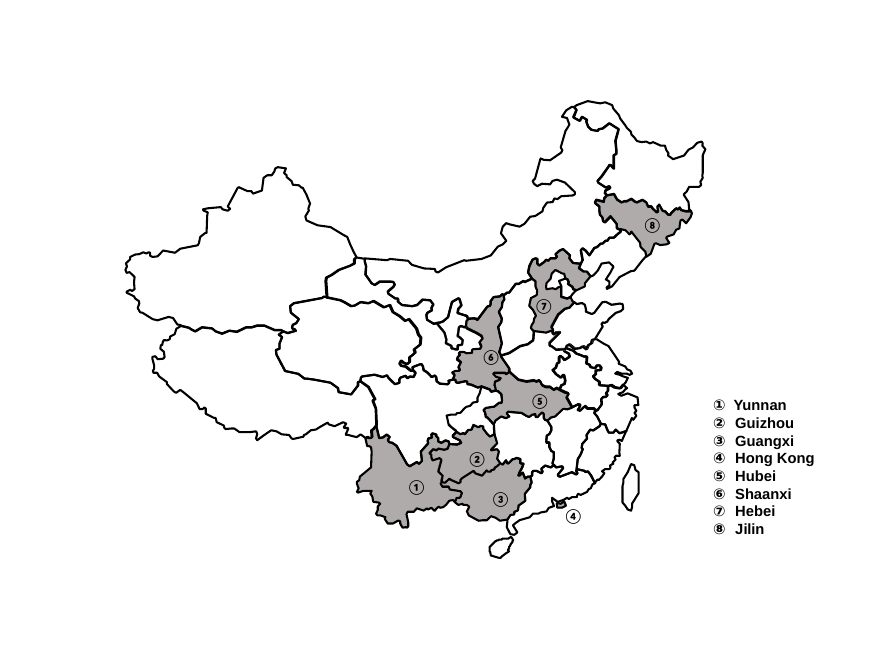

⑧
⑦
⑥
⑤
① Yunnan
Guizhou
Guangxi
Hong Kong
Hubei
Shaanxi
Hebei
Jilin
②
①
③
④

Supplement: S6 Fig — (PPTX) [file ppat.1006698.s006.pptx]

## Slide 1
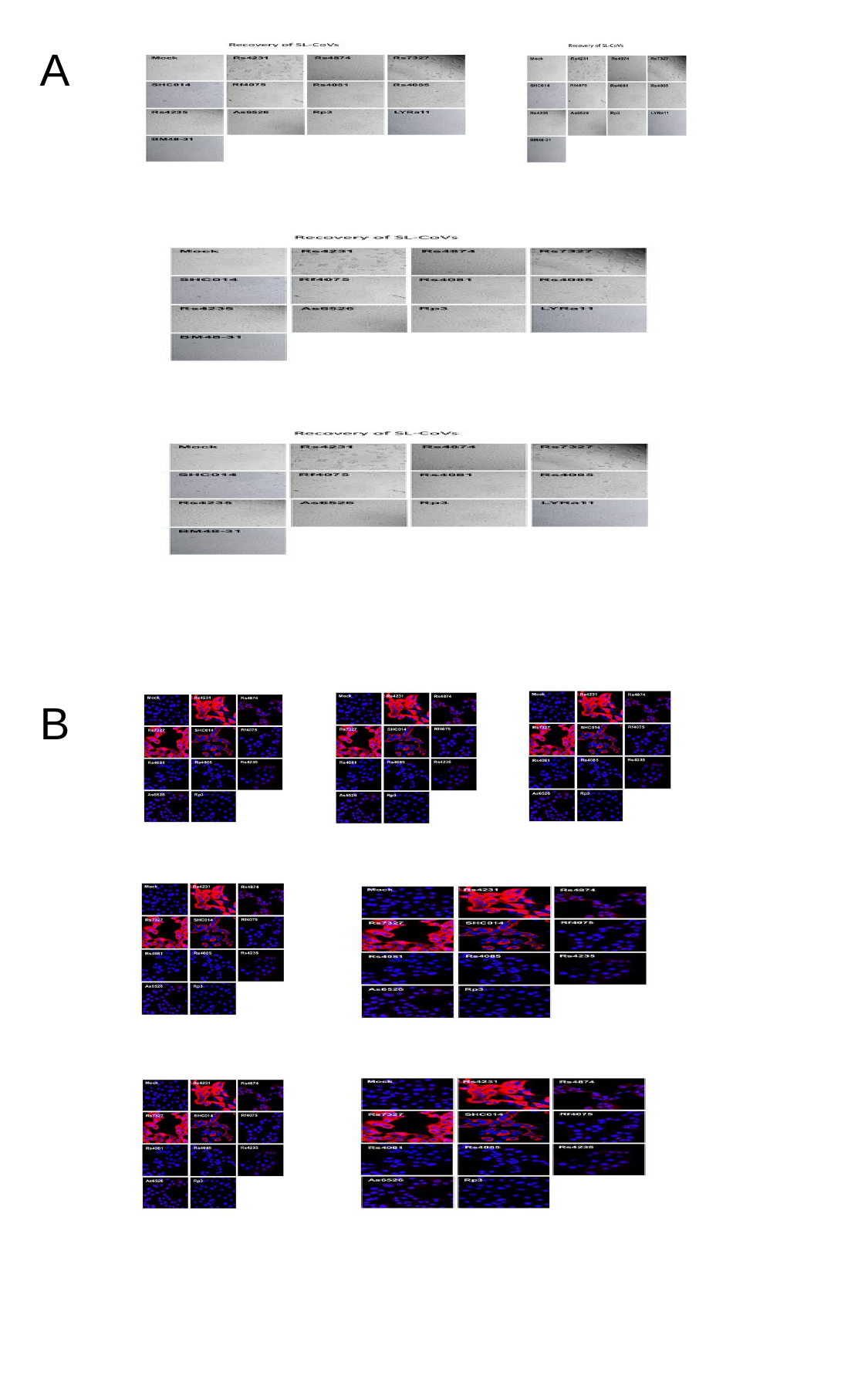

A
B

Supplement: S7 Fig — (A) Cytopathic effects in Vero E6 cells transfected with the infectious BAC clones constructed with the backbone of WIV1 and various S genes of different bat SARSr-CoV strains. Microphotographs were taken 24 hours post transfection. (B) The culture media supernatant collected from the cells transfected with the infectious BAC clones was used to infect Vero E6 cells. Immunofluorescent assay (IFA) was performed to detect infection and viral replication. Cells were fixed 24 hours post infection, and stained using rabbit antibody against the SARSr-CoV Rp3 nucleocapsid protein and a Cy3-conjugated anti-rabbit IgG. (PPTX) [file ppat.1006698.s007.pptx]

## Slide 1
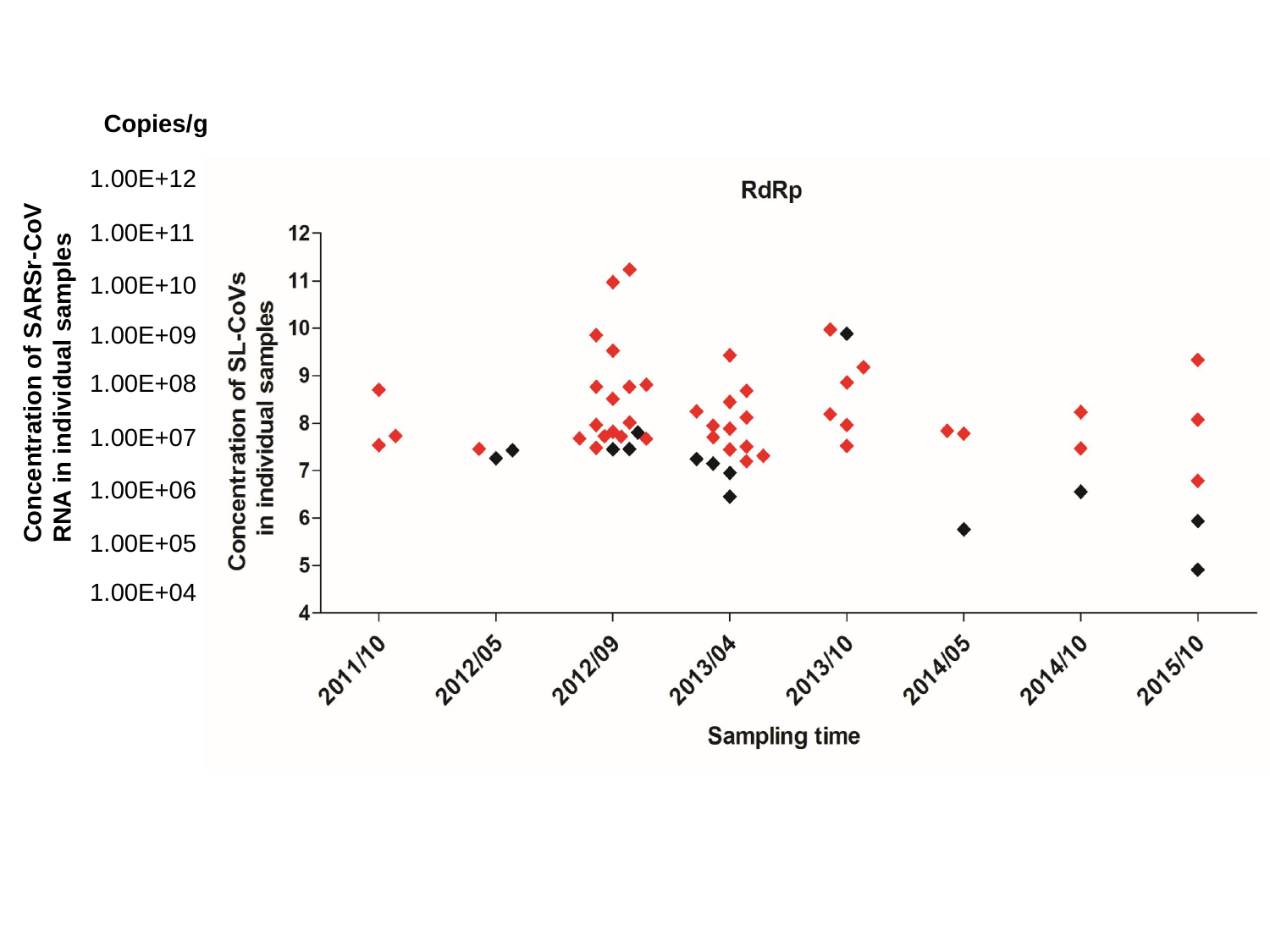

Copies/g
1.00E+12
1.00E+11
1.00E+10
1.00E+09
Concentration of SARSr-CoV RNA in individual samples
1.00E+08
1.00E+07
1.00E+06
1.00E+05
1.00E+04

Supplement: S8 Fig — The number of genome copies of SARSr-CoV per gram of bat feces was determined by quantitative real-time PCR targeting the RdRp gene. Samples from which the SARSr-CoV RBD sequences were successfully amplified are indicated in red. (PPTX) [file ppat.1006698.s008.pptx]

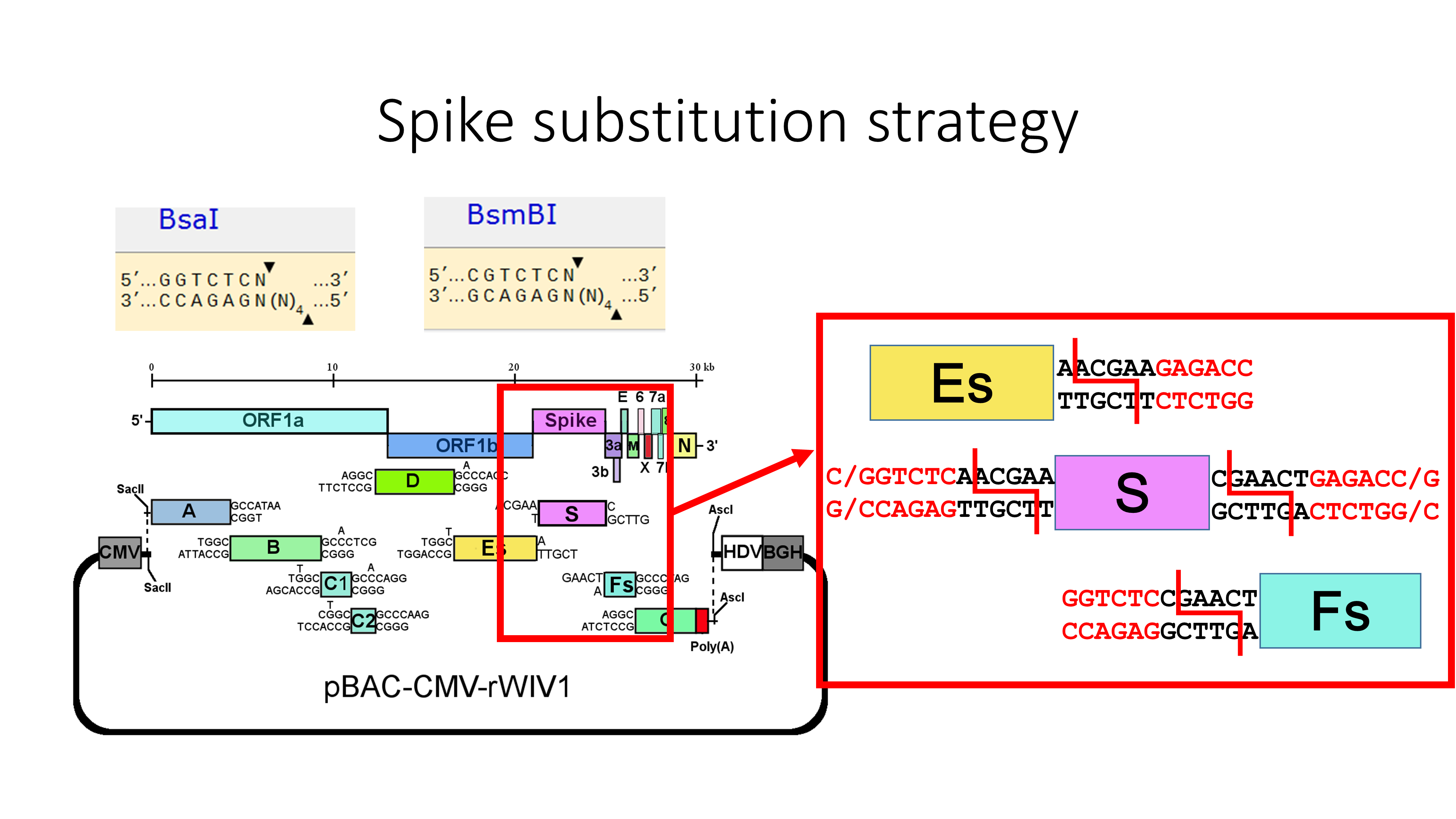

Supplement: S9 Fig — The original fragments E and F were shortened to leave spike gene as an independent fragment. The new fragments were designated as Es and Fs. BsaI or BsmBI sites were introduced into the junctions of Es/Spike and Spike/Fs. Then any spike could be substituted into the genome of SARSr-CoV WIV1 through this strategy. (TIF) [file ppat.1006698.s009.tif]
