# Supplementary material for: Discovery of a rich gene pool of bat SARS-related coronaviruses provides new insights into the origin of SARS coronavirus
Source: PLoS Pathog. 2017 Nov 30;13(11):e1006698. doi: 10.1371/journal.ppat.1006698 (PMC5708621; doi:10.1371/journal.ppat.1006698)
Supplement: S1 Table — (DOCX) [file ppat.1006698.s010.docx]

S1 Table． Comparison of the novel bat SARSr-CoVs identified in this study with human/civet SARS-CoVs and previously described bat SARSr-CoVs

**Rs4081**

| SARS-CoV or SARSr-CoV | % Amino acid identity | | | | | | | | | | | | | |
| --- | --- | --- | --- | --- | --- | --- | --- | --- | --- | --- | --- | --- | --- | --- |
|  | P1a | P1b | S | S1 | S2 | ORF3a | ORF3b | E | M | ORF6 | ORF7a | ORF7b | ORF8 | N |
| Human SARS-CoV GZ02 | 98.2 | 99.4 | 80.4 | 66.8 | 96.0 | 91.6 | 96.5 | 100 | 98.2 | 98.4 | 95.9 | 93.2 | 40.4 | 99.3 |
| Human SARS-CoV BJ01 | 98.1 | 99.4 | 80.6 | 67.1 | 96.0 | 90.5 | 95.6 | 100 | 98.2 | 96.8 | 95.9 | 93.2 | N/A | 99.3 |
| Human SARS-CoV Tor2 | 98.2 | 99.4 | 80.4 | 66.8 | 96.0 | 90.5 | 95.6 | 100 | 98.2 | 98.4 | 95.9 | 93.2 | N/A | 99.3 |
| Civet SARS-CoV SZ3 | 98.2 | 99.4 | 80.2 | 66.8 | 95.5 | 90.1 | 94.7 | 100 | 97.7 | 98.4 | 95.9 | 93.2 | 40.4 | 99.3 |
| Bat SARSr-CoV WIV1 | 99.1 | 99.6 | 80.6 | 67.3 | 96.0 | 91.2 | 95.6 | 100 | 99.1 | 95.2 | 99.2 | 100 | 99.2 | 99.1 |
| Bat SARSr-CoV WIV16 | 99.8 | 99.4 | 80.8 | 67.6 | 96.0 | 91.6 | 95.6 | 100 | 99.1 | 93.7 | 99.2 | 100 | 98.3 | 99.3 |
| Bat SARSr-CoV Rs3367 | 99.1 | 99.6 | 80.6 | 67.3 | 95.8 | 91.6 | 95.6 | 100 | 99.1 | 98.4 | 100 | 100 | 99.2 | 99.3 |
| Bat SARSr-CoV RsSHC014 | 99.2 | 99.6 | 80.3 | 66.7 | 96.0 | 91.6 | 96.5 | 98.7 | 99.1 | 98.4 | 100 | 100 | 100 | 99.8 |
| Bat SARSr-CoV LYRa11 | 95.6 | 99.1 | 81.0 | 66.4 | 97.7 | 85.0 | N/A | 98.7 | 95.9 | 93.7 | 94.3 | 90.9 | 83.5 | 97.4 |
| Bat SARSr-CoV Rs672 | 98.7 | 99.4 | 99.0 | 98.9 | 99.0 | 97.8 | 96.5 | 100 | 99.5 | 100 | 100 | 100 | 98.3 | 98.3 |
| Bat SARSr-CoV HKU3-1 | 94.4 | 98.6 | 91.1 | 86.6 | 96.3 | 88.7 | N/A | 100 | 96.8 | 92.1 | 97.5 | 95.5 | 87.6 | 96.2 |
| Bat SARSr-CoV Rp3 | 97.3 | 99.3 | 92.6 | 87.7 | 98.3 | 90.5 | N/A | 100 | 98.2 | 93.7 | 99.2 | 100 | 87.6 | 97.6 |
| Bat SARSr-CoV Rm1 | 93.7 | 98.7 | 92.2 | 86.8 | 98.4 | 89.8 | N/A | 98.7 | 96.4 | 93.7 | 97.5 | 100 | 89.3 | 97.4 |
| Bat SARSr-CoV Rf1 | 94.4 | 98.6 | 87.7 | 81.7 | 94.6 | 92.3 | 93.9 | 96.1 | 98.6 | 95.2 | 95.1 | 97.7 | 34.8 | 95.2 |
| Bat SARSr-CoV YNLF_31C | 96.7 | 99.4 | 87.8 | 81.5 | 95.0 | 93.4 | N/A | 100 | 99.5 | 90.5 | 95.9 | 100 | 36.5 | 97.9 |
| Bat SARSr-CoV JL2012 | 93.6 | 98.4 | 81.8 | 71.0 | 94.3 | 92.0 | 93.0 | 97.4 | 98.2 | 88.9 | 91.8 | 100 | N/A | 95.7 |

**Rs4231**

| SARS-CoV or SARSr-CoV | % Amino acid identity | | | | | | | | | | | | | |
| --- | --- | --- | --- | --- | --- | --- | --- | --- | --- | --- | --- | --- | --- | --- |
|  | P1a | P1b | S | S1 | S2 | ORF3a | ORF3b | E | M | ORF6 | ORF7a | ORF7b | ORF8 | N |
| Human SARS-CoV GZ02 | 98.3 | 99.4 | 95.0 | 91.6 | 99.0 | 97.8 | 97.4 | 98.7 | 99.1 | 96.8 | 95.9 | 93.2 | 38.6 | 99.5 |
| Human SARS-CoV BJ01 | 98.2 | 99.4 | 94.8 | 91.3 | 99.0 | 96.7 | 96.5 | 98.7 | 99.1 | 95.2 | 95.9 | 93.2 | N/A | 99.5 |
| Human SARS-CoV Tor2 | 98.3 | 99.4 | 94.6 | 90.9 | 99.0 | 96.7 | 96.5 | 98.7 | 99.1 | 96.8 | 95.9 | 93.2 | N/A | 99.5 |
| Civet SARS-CoV SZ3 | 98.3 | 99.4 | 94.9 | 91.8 | 98.6 | 96.4 | 95.6 | 98.7 | 98.6 | 96.8 | 95.9 | 93.2 | 38.6 | 99.5 |
| Bat SARSr-CoV WIV1 | 99.0 | 99.5 | 90.6 | 83.1 | 99.5 | 97.8 | 96.5 | 98.7 | 97.3 | 93.7 | 99.2 | 100 | 99.2 | 99.3 |
| Bat SARSr-CoV WIV16 | 99.2 | 99.6 | 96.7 | 94.3 | 99.5 | 98.2 | 96.5 | 98.7 | 97.3 | 92.1 | 99.2 | 100 | 100 | 99.5 |
| Bat SARSr-CoV Rs3367 | 99.0 | 99.4 | 90.7 | 83.4 | 99.3 | 98.2 | 96.5 | 98.7 | 97.3 | 96.8 | 100 | 100 | 99.2 | 99.5 |
| Bat SARSr-CoV RsSHC014 | 99.1 | 99.5 | 93.3 | 88.1 | 99.5 | 98.2 | 97.4 | 97.4 | 97.3 | 96.8 | 100 | 100 | 98.3 | 99.5 |
| Bat SARSr-CoV LYRa11 | 95.7 | 99.1 | 88.0 | 81.0 | 96.3 | 92.0 | N/A | 97.4 | 96.8 | 92.1 | 94.3 | 90.9 | 82.6 | 97.6 |
| Bat SARSr-CoV Rs672 | 98.7 | 99.4 | 80.1 | 66.8 | 95.3 | 90.5 | 97.4 | 98.7 | 97.7 | 95.2 | 100 | 100 | 97.5 | 98.6 |
| Bat SARSr-CoV HKU3-1 | 94.4 | 98.7 | 80.3 | 68.2 | 94.1 | 82.5 | N/A | 98.7 | 97.7 | 92.1 | 97.5 | 95.5 | 86.8 | 96.4 |
| Bat SARSr-CoV Rp3 | 97.2 | 99.3 | 80.8 | 67.3 | 96.3 | 84.3 | N/A | 98.7 | 96.4 | 90.5 | 99.2 | 100 | 86.8 | 97.9 |
| Bat SARSr-CoV Rm1 | 93.8 | 98.7 | 81.1 | 68.2 | 95.8 | 84.3 | N/A | 97.4 | 96.8 | 90.5 | 97.5 | 100 | 88.4 | 97.6 |
| Bat SARSr-CoV Rf1 | 94.2 | 98.4 | 78.9 | 67.0 | 92.7 | 86.5 | 94.7 | 94.7 | 96.8 | 93.7 | 95.1 | 97.7 | 33.0 | 95.5 |
| Bat SARSr-CoV YNLF_31C | 96.7 | 99.3 | 79.8 | 67.9 | 93.4 | 88.0 | N/A | 98.7 | 97.7 | 88.9 | 95.9 | 100 | 34.8 | 98.6 |
| Bat SARSr-CoV JL2012 | 93.4 | 98.3 | 76.8 | 62.7 | 92.3 | 86.5 | 93.9 | 98.7 | 96.4 | 87.3 | 91.8 | 100 | N/A | 96.0 |

**Rs7327**

| SARS-CoV or SARSr-CoV |  | % Amino acid identity | | | | | | | | | | | | | |
| --- | --- | --- | --- | --- | --- | --- | --- | --- | --- | --- | --- | --- | --- | --- | --- |
|  | P1a | P1b | S | S1 | S2 | ORF3a | ORF3b | E | M | ORF6 | ORFX | ORF7a | ORF7b | ORF8 | N |
| Human SARS-CoV GZ02 | 98.3 | 99.3 | 92.7 | 86.9 | 99.5 | 98.9 | 99.4 | 100 | 98.2 | 93.7 | N/A | 95.1 | 93.2 | 38.6 | 99.5 |
| Human SARS-CoV BJ01 | 98.2 | 99.3 | 92.6 | 86.8 | 99.5 | 97.8 | 97.4 | 100 | 98.2 | 92.1 | N/A | 95.1 | 93.2 | N/A | 99.5 |
| Human SARS-CoV Tor2 | 98.3 | 99.3 | 92.4 | 86.5 | 99.5 | 97.8 | 98.1 | 100 | 98.2 | 93.7 | N/A | 95.1 | 93.2 | N/A | 99.5 |
| Civet SARS-CoV SZ3 | 98.3 | 99.3 | 92.5 | 86.9 | 99.1 | 97.4 | 97.4 | 100 | 97.7 | 93.7 | N/A | 95.1 | 93.2 | 38.6 | 99.5 |
| Bat SARSr-CoV WIV1 | 99.1 | 99.8 | 99.1 | 98.7 | 99.7 | 98.9 | 98.2 | 100 | 100 | 100 | 94.8 | 98.4 | 100 | 99.2 | 99.3 |
| Bat SARSr-CoV WIV16 | 99.2 | 99.2 | 93.1 | 87.5 | 99.7 | 99.3 | 98.2 | 100 | 100 | 98.4 | 94.8 | 98.4 | 100 | 98.3 | 99.5 |
| Bat SARSr-CoV Rs3367 | 99.1 | 99.7 | 99.0 | 98.7 | 99.5 | 99.3 | 98.2 | 100 | 100 | 96.8 | N/A | 99.2 | 100 | 99.2 | 99.5 |
| Bat SARSr-CoV RsSHC014 | 98.9 | 99.8 | 97.0 | 94.7 | 99.7 | 99.3 | 99.1 | 98.7 | 100 | 96.8 | N/A | 99.2 | 100 | 98.3 | 99.5 |
| Bat SARSr-CoV LYRa11 | 95.7 | 99.1 | 89.6 | 83.8 | 96.5 | 92.0 | N/A | 98.7 | 95.9 | 92.1 | N/A | 93.4 | 90.9 | 82.6 | 97.6 |
| Bat SARSr-CoV Rs672 | 98.7 | 99.3 | 80.2 | 66.8 | 95.5 | 90.9 | 97.4 | 100 | 99.5 | 95.2 | N/A | 99.2 | 100 | 97.5 | 98.6 |
| Bat SARSr-CoV HKU3-1 | 94.4 | 98.6 | 79.9 | 67.8 | 93.9 | 82.5 | N/A | 100 | 96.8 | 88.9 | N/A | 96.7 | 95.5 | 86.0 | 96.4 |
| Bat SARSr-CoV Rp3 | 97.1 | 99.2 | 80.7 | 67.3 | 96.2 | 84.3 | N/A | 100 | 98.2 | 90.5 | N/A | 98.4 | 100 | 86.0 | 97.9 |
| Bat SARSr-CoV Rm1 | 93.8 | 98.7 | 80.9 | 67.7 | 96.0 | 84.3 | N/A | 98.7 | 95.5 | 90.5 | N/A | 96.7 | 100 | 87.6 | 97.6 |
| Bat SARSr-CoV Rf1 | 94.3 | 98.5 | 78.9 | 66.8 | 92.9 | 86.9 | 91.2 | 96.1 | 98.6 | 90.5 | N/A | 94.3 | 97.7 | 33.0 | 95.5 |
| Bat SARSr-CoV YNLF_31C | 96.7 | 99.4 | 80.0 | 67.9 | 93.9 | 88.3 | N/A | 100 | 99.5 | 92.1 | N/A | 95.1 | 100 | 34.8 | 98.1 |
| Bat SARSr-CoV JL2012 | 93.5 | 98.2 | 76.8 | 62.8 | 92.5 | 86.9 | 90.4 | 97.4 | 98.6 | 84.1 | N/A | 91.0 | 100 | N/A | 96.0 |

**Rs4874**

| SARS-CoV or SARSr-CoV |  | % Amino acid identity | | | | | | | | | | | | | |
| --- | --- | --- | --- | --- | --- | --- | --- | --- | --- | --- | --- | --- | --- | --- | --- |
|  | P1a | P1b | S | S1 | S2 | ORF3a | ORF3b | E | M | ORF6 | ORFX | ORF7a | ORF7b | ORF8 | N |
| Human SARS-CoV GZ02 | 98.1 | 99.1 | 97.4 | 95.6 | 99.5 | 98.2 | 99.1 | 100 | 97.7 | 93.7 | N/A | 95.1 | 93.2 | 38.6 | 99.5 |
| Human SARS-CoV BJ01 | 98.0 | 99.1 | 97.2 | 95.3 | 99.5 | 97.1 | 98.2 | 100 | 97.7 | 92.1 | N/A | 95.1 | 93.2 | N/A | 99.5 |
| Human SARS-CoV Tor2 | 98.1 | 99.1 | 97.0 | 94.9 | 99.5 | 97.1 | 98.2 | 100 | 97.7 | 93.7 | N/A | 95.1 | 93.2 | N/A | 99.5 |
| Civet SARS-CoV SZ3 | 98.1 | 99.1 | 97.2 | 95.6 | 99.1 | 96.7 | 97.4 | 100 | 97.3 | 93.7 | N/A | 95.1 | 93.2 | 38.6 | 99.5 |
| Bat SARSr-CoV WIV1 | 99.0 | 99.2 | 93.9 | 88.7 | 100 | 99.6 | 100 | 100 | 99.5 | 100 | 94.8 | 100 | 100 | 99.2 | 99.3 |
| Bat SARSr-CoV WIV16 | 100.0 | 99.7 | 99.9 | 99.9 | 100 | 100 | 100 | 100 | 99.5 | 98.4 | 94.8 | 100 | 100 | 100 | 100 |
| Bat SARSr-CoV Rs3367 | 99.0 | 99.1 | 93.9 | 89.0 | 99.8 | 100 | 100 | 100 | 99.5 | 96.8 | N/A | 99.2 | 100 | 99.2 | 99.5 |
| Bat SARSr-CoV RsSHC014 | 99.0 | 99.2 | 91.1 | 83.5 | 100 | 100 | 99.1 | 98.7 | 99.5 | 96.8 | N/A | 99.2 | 100 | 98.3 | 99.5 |
| Bat SARSr-CoV LYRa11 | 95.5 | 98.8 | 90.4 | 85.1 | 96.5 | 92.0 | N/A | 98.7 | 95.5 | 92.1 | N/A | 93.4 | 90.9 | 82.6 | 97.9 |
| Bat SARSr-CoV Rs672 | 98.6 | 99.1 | 80.6 | 67.6 | 95.5 | 90.9 | 97.4 | 100 | 99.1 | 95.2 | N/A | 99.2 | 100 | 97.5 | 98.6 |
| Bat SARSr-CoV HKU3-1 | 94.3 | 98.4 | 80.2 | 68.2 | 93.9 | 82.5 | N/A | 100 | 96.4 | 88.9 | N/A | 96.7 | 95.5 | 86.8 | 96.7 |
| Bat SARSr-CoV Rp3 | 97.2 | 99.0 | 81.1 | 67.7 | 96.5 | 84.3 | N/A | 100 | 97.7 | 90.5 | N/A | 98.4 | 100 | 86.8 | 97.9 |
| Bat SARSr-CoV Rm1 | 93.6 | 98.4 | 81.4 | 68.6 | 96.0 | 84.3 | N/A | 98.7 | 95.0 | 90.5 | N/A | 96.7 | 100 | 88.4 | 97.6 |
| Bat SARSr-CoV Rf1 | 94.3 | 98.2 | 79.5 | 67.6 | 93.2 | 86.9 | 91.2 | 96.1 | 98.2 | 90.5 | N/A | 94.3 | 97.7 | 33.0 | 95.5 |
| Bat SARSr-CoV YNLF_31C | 96.6 | 99.0 | 80.3 | 68.5 | 93.9 | 88.3 | N/A | 100 | 99.1 | 92.1 | N/A | 95.1 | 100 | 34.8 | 98.1 |
| Bat SARSr-CoV JL2012 | 93.5 | 98.0 | 77.3 | 63.2 | 92.9 | 86.9 | 90.4 | 97.4 | 98.2 | 84.1 | N/A | 91.0 | 100 | N/A | 96.0 |

**Rs4084**

| SARS-CoV or SARSr-CoV |  | % Amino acid identity | | | | | | | | | | | | | |
| --- | --- | --- | --- | --- | --- | --- | --- | --- | --- | --- | --- | --- | --- | --- | --- |
|  | P1a | P1b | S | S1 | S2 | ORF3a | ORF3b | E | M | ORF6 | ORF7a | ORF7b | ORF8a | ORF8b | N |
| Human SARS-CoV GZ02 | 98.1 | 99.0 | 90.1 | 82.2 | 99.5 | 98.2 | 100 | 98.7 | 98.2 | 96.8 | 95.9 | 93.2 | N/A | N/A | 99.5 |
| Human SARS-CoV BJ01 | 98.0 | 99.0 | 90.0 | 82.1 | 99.5 | 97.1 | 99.1 | 98.7 | 98.2 | 95.2 | 95.9 | 93.2 | 100 | 96.4 | 99.5 |
| Human SARS-CoV Tor2 | 98.0 | 98.9 | 89.9 | 81.8 | 99.5 | 97.1 | 99.1 | 98.7 | 98.2 | 96.8 | 95.9 | 93.2 | 97.4 | 96.4 | 99.5 |
| Civet SARS-CoV SZ3 | 98.1 | 99.0 | 90.0 | 82.4 | 99.1 | 96.7 | 98.2 | 98.7 | 97.7 | 96.8 | 95.9 | 93.2 | N/A | N/A | 99.5 |
| Bat SARSr-CoV WIV1 | 98.9 | 99.6 | 97.0 | 94.4 | 100 | 99.6 | 99.1 | 98.7 | 100 | 93.7 | 99.2 | 100 | N/A | N/A | 99.3 |
| Bat SARSr-CoV WIV16 | 99.1 | 98.8 | 90.9 | 83.2 | 100 | 100 | 99.1 | 98.7 | 100 | 92.1 | 99.2 | 100 | N/A | N/A | 99.1 |
| Bat SARSr-CoV Rs3367 | 98.9 | 99.6 | 96.9 | 94.4 | 99.8 | 100 | 99.1 | 98.7 | 100 | 96.8 | 100 | 100 | N/A | N/A | 99.5 |
| Bat SARSr-CoV RsSHC014 | 99.1 | 99.7 | 99.9 | 99.9 | 100 | 100 | 100 | 100 | 100 | 96.8 | 100 | 100 | N/A | N/A | 99.1 |
| Bat SARSr-CoV LYRa11 | 95.6 | 98.8 | 87.0 | 79.0 | 96.5 | 92.0 | N/A | 97.4 | 95.9 | 92.1 | 94.3 | 90.9 | N/A | N/A | 97.4 |
| Bat SARSr-CoV Rs672 | 98.6 | 98.9 | 79.9 | 66.4 | 95.5 | 90.9 | 98.2 | 98.7 | 99.5 | 95.2 | 100 | 100 | N/A | N/A | 98.1 |
| Bat SARSr-CoV HKU3-1 | 94.4 | 98.3 | 79.8 | 67.5 | 93.9 | 82.5 | N/A | 98.7 | 96.8 | 92.1 | 97.5 | 95.5 | N/A | N/A | 96.2 |
| Bat SARSr-CoV Rp3 | 97.2 | 98.9 | 80.8 | 67.1 | 96.5 | 84.3 | N/A | 98.7 | 98.2 | 90.5 | 99.2 | 100 | N/A | N/A | 97.6 |
| Bat SARSr-CoV Rm1 | 93.7 | 98.4 | 80.8 | 67.6 | 96.0 | 84.3 | N/A | 97.4 | 95.5 | 90.5 | 97.5 | 100 | N/A | N/A | 97.4 |
| Bat SARSr-CoV Rf1 | 94.2 | 98.1 | 79.0 | 66.7 | 93.2 | 86.9 | 92.1 | 96.1 | 98.6 | 90.5 | 95.1 | 97.7 | N/A | N/A | 95.2 |
| Bat SARSr-CoV YNLF_31C | 96.6 | 99.1 | 79.9 | 67.7 | 93.9 | 88.3 | N/A | 98.7 | 99.5 | 88.9 | 95.9 | 100 | N/A | N/A | 97.9 |
| Bat SARSr-CoV JL2012 | 93.4 | 97.9 | 76.9 | 62.7 | 92.9 | 86.9 | 91.2 | 96.1 | 98.6 | 84.1 | 91.8 | 100 | N/A | N/A | 95.7 |

**Rf4092**

| SARS-CoV or SARSr-CoV | % Amino acid identity | | | | | | | | | | | | | |
| --- | --- | --- | --- | --- | --- | --- | --- | --- | --- | --- | --- | --- | --- | --- |
|  | P1a | P1b | S | S1 | S2 | ORF3a | ORF3b | E | M | ORF6 | ORF7a | ORF7b | ORF8 | N |
| Human SARS-CoV GZ02 | 98.1 | 99.2 | 78.5 | 63.3 | 95.3 | 90.9 | 95.6 | 100 | 98.6 | 98.4 | 95.9 | 93.2 | 97.5 | 99.3 |
| Human SARS-CoV BJ01 | 97.9 | 99.2 | 78.6 | 63.5 | 95.3 | 89.8 | 94.7 | 100 | 98.6 | 96.8 | 95.9 | 93.2 | N/A | 99.3 |
| Human SARS-CoV Tor2 | 98.0 | 99.2 | 78.5 | 63.3 | 95.3 | 89.8 | 94.7 | 100 | 98.6 | 98.4 | 95.9 | 93.2 | N/A | 99.3 |
| Civet SARS-CoV SZ3 | 98.1 | 99.2 | 78.2 | 63.3 | 94.8 | 89.4 | 93.9 | 100 | 98.2 | 98.4 | 95.9 | 93.2 | 97.5 | 99.3 |
| Bat SARSr-CoV WIV1 | 98.7 | 99.4 | 78.3 | 63.1 | 95.3 | 91.2 | 94.7 | 100 | 99.5 | 95.2 | 99.2 | 97.7 | 38.6 | 99.1 |
| Bat SARSr-CoV WIV16 | 98.8 | 99.1 | 78.5 | 63.3 | 95.3 | 91.6 | 94.7 | 100 | 99.5 | 93.7 | 99.2 | 97.7 | 37.7 | 98.8 |
| Bat SARSr-CoV Rs3367 | 98.7 | 99.4 | 78.3 | 63.3 | 95.1 | 91.6 | 94.7 | 100 | 99.5 | 98.4 | 100 | 97.7 | 38.6 | 99.3 |
| Bat SARSr-CoV RsSHC014 | 98.6 | 99.4 | 78.3 | 63.1 | 95.3 | 91.6 | 95.6 | 98.7 | 99.5 | 98.4 | 100 | 97.7 | 39.5 | 98.8 |
| Bat SARSr-CoV LYRa11 | 95.4 | 99.0 | 79.7 | 63.5 | 97.7 | 85.0 | N/A | 98.7 | 96.4 | 93.7 | 94.3 | 93.2 | 37.7 | 97.2 |
| Bat SARSr-CoV Rs672 | 98.4 | 99.2 | 85.0 | 72.5 | 99.5 | 97.1 | 93.9 | 100 | 100 | 96.8 | 100 | 97.7 | 38.6 | 97.9 |
| Bat SARSr-CoV HKU3-1 | 94.1 | 98.5 | 83.5 | 72.4 | 96.2 | 87.6 | N/A | 100 | 97.3 | 93.7 | 97.5 | 95.5 | 36.8 | 96.2 |
| Bat SARSr-CoV Rp3 | 96.9 | 99.1 | 84.5 | 72.7 | 98.1 | 90.5 | N/A | 100 | 98.6 | 92.1 | 99.2 | 97.7 | 38.6 | 97.4 |
| Bat SARSr-CoV Rm1 | 93.5 | 98.6 | 84.1 | 71.8 | 98.3 | 90.5 | N/A | 98.7 | 95.9 | 92.1 | 97.5 | 97.7 | 38.6 | 97.1 |
| Bat SARSr-CoV Rf1 | 94.1 | 98.4 | 82.2 | 72.1 | 93.9 | 93.1 | 91.2 | 96.1 | 99.1 | 92.1 | 95.1 | 95.5 | 83.5 | 95.0 |
| Bat SARSr-CoV YNLF_31C | 96.5 | 99.3 | 82.0 | 71.3 | 94.3 | 92.0 | N/A | 100 | 100 | 90.5 | 95.9 | 97.7 | 84.3 | 97.6 |
| Bat SARSr-CoV JL2012 | 93.2 | 98.1 | 92.1 | 90.9 | 93.6 | 91.6 | 90.4 | 97.4 | 98.6 | 85.7 | 91.8 | 97.7 | N/A | 95.5 |

*S1, the N-terminal domain of the coronavirus S protein responsible for receptor binding. S2, the S protein C-terminal domain responsible for membrane fusion. The ORFs in the genome were predicted and putative protein sequences were translated. The pairwise comparisons were conducted for all ORFs at amino acids (aa) levels. N/A, not available.
