# Supplementary material for: Discovery of a rich gene pool of bat SARS-related coronaviruses provides new insights into the origin of SARS coronavirus
Source: PLoS Pathog. 2017 Nov 30;13(11):e1006698. doi: 10.1371/journal.ppat.1006698 (PMC5708621; doi:10.1371/journal.ppat.1006698)
Supplement: S2 Table — (DOCX) [file ppat.1006698.s011.docx]

S2 Table Distribution of SARSr-CoVs highly similar to SARS-CoV in the variable S, ORF3 and ORF8 genes in the single cave

| Gene | SARSr-CoV strains highly similar to SARS-CoV | Sampling time  (No. of strains) | Bat species  (No. of strains) |
| --- | --- | --- | --- |
| S-RBD | **WIV1**, **Rs3367**, Rs4079, Rs4087, Rs4090, Rs4105, Rs4230, Rs4829, Rs4832, **WIV16**, **Rs4874**, Rs4952,  Rs7326, **Rs7327**, **Rs9401**, Rs9403 | May 2012 (2)  Sep 2012 (4)  Apr 2013 (1)  Jul 2013 (5)  Oct 2014 (2)  Oct 2015 (2) | *Rhinolophus sinicus* (16) |
| S-NTD | **Rs4231**, **WIV16**, **Rs4874**, Rs4952 | Apr 2013 (1)  Jul 2013 (3) | *Rhinolophus sinicus* (4) |
| ORF8 | Rs3261, Rf4075, **Rs4084**, Rs4091, **Rf4092**, Rs4110, Rf4122, Rs4832, Rs4943 | Oct 2011 (1)  Sep 2012 (6)  Jul 2013 (2) | *Rhinolophus sinicus* (6)  *Rhinolophus ferrumequinum* (3) |
| ORF3a | **RsSHC014**, **WIV1**, **Rs3367**, Rs3369, **Rs4084**, Rs4087, Rs4090, Rs4105, **Rs4231**, Rs4829, **WIV16**, **Rs4874**, Rs4900, Rs4952, Rs7326, **Rs7327**, **Rs9401** | Apr 2011 (1)  May 2012 (3)  Sep 2012 (4)  Apr 2013 (1)  Jul 2013 (5)  Oct 2014 (2)  Oct 2015 (1) | *Rhinolophus sinicus* (17) |
| ORF3b | Rs7326, **Rs7327** | Oct 2014 (2) | *Rhinolophus sinicus* (2) |
